# Exposure to environmental radionuclides is associated with altered metabolic and immunity pathways in a wild rodent

**DOI:** 10.1111/mec.15241

**Published:** 2019-09-30

**Authors:** Jenni Kesäniemi, Toni Jernfors, Anton Lavrinienko, Kati Kivisaari, Mikko Kiljunen, Tapio Mappes, Phillip C. Watts

**Affiliations:** ^1^ Ecology and Genetics Research Unit University of Oulu Oulu Finland; ^2^ Department of Biological and Environmental Science University of Jyväskylä Jyväskylä Finland

**Keywords:** DNA repair, *Myodes glareolus*, pollution, radionuclides, RNAseq, stable isotope

## Abstract

Wildlife inhabiting environments contaminated by radionuclides face putative detrimental effects of exposure to ionizing radiation, with biomarkers such as an increase in DNA damage and/or oxidative stress commonly associated with radiation exposure. To examine the effects of exposure to radiation on gene expression in wildlife, we conducted a de novo RNA sequencing study of liver and spleen tissues from a rodent, the bank vole *Myodes glareolus*. Bank voles were collected from the Chernobyl Exclusion Zone (CEZ), where animals were exposed to elevated levels of radionuclides, and from uncontaminated areas near Kyiv, Ukraine. Counter to expectations, we did not observe a strong DNA damage response in animals exposed to radionuclides, although some signs of oxidative stress were identified. Rather, exposure to environmental radionuclides was associated with upregulation of genes involved in lipid metabolism and fatty acid oxidation in the livers – an apparent shift in energy metabolism. Moreover, using stable isotope analysis, we identified that fur from bank voles inhabiting the CEZ had enriched isotope values of nitrogen: such an increase is consistent with increased fatty acid metabolism, but also could arise from a difference in diet or habitat between the CEZ and elsewhere. In livers and spleens, voles inhabiting the CEZ were characterized by immunosuppression, such as impaired antigen processing, and activation of leucocytes involved in inflammatory responses. In conclusion, exposure to low dose environmental radiation impacts pathways associated with immunity and lipid metabolism, potentially as a stress‐induced coping mechanism.

## 1 INTRODUCTION

Human actions pose numerous stressors such as warming, extreme weather and pollutants, to wildlife at both global and local scales, with loss and deterioration of habitat presenting major impacts to many species (Acevedo‐Whitehouse & Duffus, 2009). Organisms that persist in the face of a changing environment need to mount an appropriate genomic response, often achieved using altered transcriptional activity. Analyses of these transcriptional changes provide key insights into the biological pathways that underline any physiological response (Evans, Pespeni, Hofmann, Palumbi, & Sanford, 2017; Pujolar et al., 2012).

Exposure to radionuclides is a source of genotoxicity to wildlife, with numerous human activities, for example processes involved in uranium mining, nuclear energy production and its waste treatment, or nuclear tests, having left many contaminated areas worldwide (reviewed by Lourenço, Mendo, & Pereira, 2016). Perhaps the most notable areas of radionuclide contamination in the natural environment are derived from the accidents at the Nuclear Power Plants (NPPs) at Chernobyl (Ukraine, 1986) and at Fukushima (Japan, 2011). Wildlife inhabiting the area surrounding the former NPP at Chernobyl provide the best‐studied models of the biological impacts of exposure to environmental radionuclides. The accident at the Chernobyl NPP Reactor 4 released more than 9 million terabecquerels (TBq) of radionuclides over a wide area (>200,000 km^2^) of Europe and eastern Russia. Subsequently, the Chernobyl Exclusion Zone (CEZ) was established at an approximately 30 km radius (~4,300 km^2^ area) around the accident site to limit human exposure to the radioactive fallout. However, the wildlife inhabiting the CEZ are exposed to elevated levels of persistent radioisotopes, notably strontium‐90 (^90^Sr), cesium‐137 (^137^Cs), and plutonium‐239 (^239^Pu) (Møller & Mousseau, 2006).

Detrimental effects of chronic exposure to radiation in wildlife have been reported at multiple biological scales (reviewed by Lourenço et al., 2016). For example, the community diversity of soil bacteria (Romanovskaya, Sokolov, Rokitko, & Chernaya, 1998), abundance of soil invertebrates (Møller & Mousseau, 2018) and the density of mammals (Møller & Mousseau, 2013) negatively correlate with levels of radiation within and around the CEZ. At the organismal level, wildlife affected by Chernobyl fallout exhibit a suite of phenotypic effects such as aspermy and reduced sperm motility (Møller, Bonisoli‐Alquati, Mousseau, & Rudolfsen, 2014) and smaller brains (Møller, Bonisoli‐Alquati, Rudolfsen, & Mousseau, 2011): comparable impacts have been reported in studies of organisms affected by the Fukushima nuclear accident (Lourenço et al., 2016). Conversely, many studies fail to find notable biological impacts of exposure to environmental radionuclides, for example on the community diversity of macro‐organisms (Murphy, Nagorskaya, & Smith, 2011), or in the abundance or density of wildlife (Deryabina et al., 2015). At a molecular level, an apparent increase of DNA damage, chromosomal aberrations (Lourenço et al., 2016), oxidative stress (Einor, Bonisoli‐Alquati, Costantini, Mousseau, & Møller, 2016) and/or mutation rate (Møller & Mousseau, 2015) have been associated with exposure to environmental radionuclides (but cf. [Kesäniemi et al., 2018] who found no evidence that mutation rate was elevated in bank voles inhabiting the CEZ). Despite the numerous and often contradictory studies on the diverse impacts of exposure to environmental radionuclides, changes in genome‐wide gene expression associated with exposure to low dose environmental radionuclides are poorly understood.

While exposure to environmental radionuclides impacts gene expression, it is hard to identify a general response of organisms because studies typically quantify expression of candidate genes from DNA repair and oxidative stress pathways. For example, the level of soil radionuclides within and around the CEZ is associated with the activity of some candidate radical scavenging and DNA damage response genes in plants (Kovalchuk, 2004) and in a rodent, the bank vole *Myodes glareolus* (Jernfors et al., 2018). Away from Chernobyl, increased DNA damage and elevated expression of selected DNA damage and repair candidate genes were observed in European wood mice (*Apodemus sylvaticus*) exposed to uranium mining waste (Lourenço, Pereira, Gonçalves, & Mendo, 2013) and marine mussels (*Mytilus* sp.) from sediments with low (0.61 μGy/hr) levels of radionuclides (Alamri, Cundy, Di, Jha, & Rotchell, 2012). A clear limitation of the candidate gene approach is that it overlooks the potential action of many other cellular and molecular processes. For example, biomedical studies have shown that in addition to inducing DNA repair pathways, exposure to acute, high dose (>1 Gy) radiation can repress the adaptive immune system while stimulating a proinflammatory response (Di Maggio et al., 2015; Hekim, Cetin, Nikitaki, Cort, & Saygili, 2015). The effects of chronic exposure to relatively low environmental radiation in wildlife immunity is not well known. However, a microarray study has shown that exposure to environmental radionuclides correlates with upregulation of the inflammatory cytokine IFNγ in the intestines of pigs affected by radionuclide fallout from Fukushima (Morimoto et al., 2017). Here, we used RNAseq to analyze the transcriptional response by wildlife (the bank vole) exposed to environmental radionuclides.

The bank vole *Myodes glareolus* is a small rodent that inhabits deciduous or coniferous forests throughout much of northern Europe and Asia (Macdonald, 2007). This species is an ideal model to quantify the genomic effects of exposure to environmental radiation as it was one of the first mammals to recolonize areas contaminated by radionuclides following the Chernobyl accident (Chesser et al., 2000). As bank voles burrow in soil and have a varied diet (including fungi, invertebrates and plants; Butet & Delettre, 2011), animals living within the CEZ can experience considerable absorbed doses of radiation (either from the soil or by consuming contaminated food); for example, bank voles from the Red Forest had average absorbed doses of radiocesium of 6.7 mGy/day (Baker et al., 2017; Chesser et al., 2000).

To examine the effects of environmental radionuclide exposure on gene expression in wild bank voles, RNAseq data were obtained for the liver and spleen tissues, as they have contrasting biological functions and different radiosensitivities. The liver is an organism's metabolic centre where it regulates energy metabolism, detoxification processes and produces diverse metabolites. The spleen has a central role in maintaining immune system function, for example via its association with storage and activation of immune cells, antibody release, and production of inflammatory mediators. The spleen, like other lymphoid organs, has a high radiosensitivity, in contrast to the liver that apparently has a fairly low radiosensitivity (Rubin & Casarett, 1968). We predicted that exposure to radionuclides will (a) elicit changes in transcriptional activity of DNA repair and oxidative stress response pathway genes, given the prevalence of studies reporting the impact of exposure to radionuclides on these molecular functions (reviewed by Einor et al., 2016; Lourenço et al., 2016). Also, we expected to find (b) changes in gene expression affecting the immune and inflammatory pathways given the prominence of these pathways in biomedical literature examining molecular impacts of exposure to radiation (Hekim et al., 2015; Kam & Banati, 2013). Finally, we predicted (c) more pronounced transcriptional differences in immune responses in the spleen than in the liver, due to its role in immune functions and its apparently high radiosensitivity.

## 2 MATERIALS AND METHODS

### 2.1 Sample collection

Bank voles were collected from four sampling areas (*n* = 40, 10 voles from each area) in Ukraine (18th‐25th of July, 2016): two locations (Vesnyane and Gluboke, 33 km apart) within the Chernobyl Exclusion Zone (CEZ) that were contaminated by radionuclides, and two uncontaminated locations near Kyiv (Kyiv west and east, 26 km apart; see map in Figure S1). We sampled voles from both sides of the Dnieper and Pripyat rivers, which represent a population genetic barrier. As bank voles from locations to the east of these rivers are genetically more similar than bank voles from locations on the west of these rivers (Kesäniemi, J., Lavrinienko, A., Tukalenko, E., Boratyński, Z., Kivisaari, K., Mappes, T., Milinevsky, G., Møller, A.P., Mousseau, T.A. & Watts, P.C., unpublished data), our sampling design incorporates two genetically different samples within each treatment, while maintaining genetically similar samples among treatments (e.g., the locations Vesnyane, CEZ and Kyiv west were similar). Animals were sampled from similar mixed forest habitats.

Bank voles were trapped using the Ugglan Special2 live traps (Grahnab, Sweden), with sunflower seeds and potato as bait. Environmental radiation was measured at ground level at each trapping site using a hand‐held GM dosimeter (Inspector, International Medcom INC, Sebastopol, CA, USA). The mean level of ambient radiation dose rate (measured in µGy/hr) within the CEZ locations was significantly higher than at the locations outside the CEZ (µGy/hr, mean ± *SD*: Vesnyane = 18.03 ± 0.9, Gluboke = 14.74 ± 5.3, Kyiv west = 0.15 ± 0.0, Kyiv east = 0.30 ± 0.0; Kruskal–Wallis test, *χ* ^2^ = 34.871, *df* = 3, *p* < .001).

We estimated ^137^Cs activity (whole‐body burden) for each vole using a SAM 940 radionuclide identifier system (Berkeley Nucleonics Corporation, San Rafael, CA, USA) equipped with a 3″ × 3″ NaI detector. The detector was shielded by 10 cm of lead to reduce noise from the background radioactivity. With corrections for laboratory background, the activity of ^137^Cs was evaluated from the obtained spectra in energies window 619–707 keV with cesium photopeak at 662 keV. Reliable measurements of cesium activity could not be made for 14 voles from Kyiv, because the ^137^Cs activity in these animals was below the detectability level, i.e. the decision threshold (the level of 95% probability that the signal from detector is caused by background fluctuation). Cesium activity (radiocesium Becquerels per kg, Bq/kg) was thus estimated for six bank voles from the Kyiv locations (Bq/kg, median [lower quartile;upper quartile]: Kyiv = 542 [506; 594]) and all CEZ voles (Bq/kg, median [lower quartile; upper quartile]: Vesnyane = 4,117 [2,620; 22,215], Gluboke = 12,319 [2,797; 27,044]). Cesium activity was significantly higher in voles from the CEZ than from Kyiv locations (Mann‐Whitney *U* test, *U* = 120.00, *p* < .001, *n* = 26).

Animals were euthanized by cervical dislocation and the livers and spleens immediately stored in AllProtect Tissue Reagent (Qiagen). Body size (weight, head width) was recorded for all individuals. As a general estimation of physiological condition of the voles, a body condition index was calculated for each individual as the standardized residual value from a linear regression of weight (dependent variable) against head width: a positive body condition index value reflects a better condition, i.e., heavier animals with greater energy reserves (Schulte‐Hostedde, Millar, & Hickling, 2001).

### 2.2 RNA extraction, library preparation and sequencing

Total RNA was extracted using RNeasy Mini Kit (Qiagen) according to the manufacturer's protocol. Samples from both tissues and all sampling locations were processed (RNA extractions and sequencing) at random order (see Appendix S1). Libraries were prepared using an Illumina TruSeq RNA Sample Prep Kit version 2 for 100 bp paired‐end (PE) sequencing. Individually barcoded samples were sequenced on an Illumina HiSeq4000 (Beijing Institute of Genomics, Hong Kong).

### 2.3 De novo transcriptome assembly, annotation and data analysis

Liver and spleen reads from four voles (one from each location) were combined (a total of 156,478,452 PE reads) for de novo transcriptome assembly. The transcriptome was assembled using Trinity version 2.4.0 (Grabherr et al., 2011) with default parameters. Most downstream analyses followed Trinity best‐practice guidelines (Haas et al., 2013; see Appendix S1). The transcriptome was annotated using Trinotate version 3.0.1 (https://trinotate.github.io/). Reads from liver (*n* = 39, as sequencing of one liver RNA sample from Kyiv east failed, see Table S1) and spleen (*n* = 40) samples were mapped to the transcriptome using Bowtie version 1.1.1 (Langmead, Trapnell, Pop, & Salzberg, 2009), with number of aligned reads quantified using rsem version 1.3.0 (Li & Dewey, 2011). Transcript abundance was examined by principal component analysis, which revealed one individual (from Vesnyane, CEZ; Figure S2) to be clearly positioned outside the cluster of all other samples (in both tissues). This outlier individual was removed from all subsequent analyses (leaving final sample sizes of *n* = 38 for liver and *n* = 39 for spleen, Figure S2). Analysis of differential expression comparing CEZ sites and uncontaminated Kyiv sites for the two tissues separately were performed at the gene level using deseq2 version 1.10.1 (Love, Huber, & Anders, 2014), with a two‐fold minimum change and a maximum false discovery rate (FDR) threshold of 0.001. Biological functions and pathways affected by environmental radiation were identified using gene ontology (GO) enrichment analysis implemented by GOseq Bioconductor version 1.26.0 (Young, Wakefield, Smyth, & Oshlack, 2010) on the DE genes (DEGs) within each tissue and the assembled and annotated transcriptome as a background set.

### 2.4 Gene coexpression networks

To investigate whether inhabiting the CEZ impacted gene expression interactions, weighted topological overlap gene coexpression networks were constructed using wto (Gysi, Voigt, Fragoso, Almaas, & Nowick, 2018) in r 3.5.0 (R Core Team, 2018). For both tissues separately, pairwise correlation networks with positive and negative interactions between genes were built for each of the four sites (using wto. Complete mode with Pearson correlations and 1,000 bootstraps), using 3,000 of the most highly expressed genes in both tissues (TPM count data). Consensus networks were then built for the CEZ and Kyiv sites. To identify similarities and differences between the consensus networks, the networks were compared using CoDiNA (Gysi, de Miranda Fragoso, Buskamp, Almaas, & Nowick, 2018) in r, where only the significant correlations were retained (links with not significant wto values [*p* > .001] were set to zero). CoDiNA assigns each link into one of three categories; α links are present in both consensus networks with the same sign (negative or positive correlation between genes), β links are present in both networks but with a different sign of the link's weight (different interaction between the genes), and γ links are specific to one of the networks (see Appendix S1).

### 2.5 Stable isotope analysis

As voles from the CEZ showed differences in metabolic pathways compared to the voles from uncontaminated areas, bank vole dietary preferences were examined using stable isotope analysis (SIA). SIA from consumers' tissues reflects the isotopic composition of the diet, since food sources, such as plant types, seeds or invertebrates, differ in their isotopic compositions (Baltensperger, Huettmann, Hagelin, & Welker, 2015; Calandra et al., 2015). Animal fur has a slow isotopic turnover rate and reflects the isotopic signal of consumed food from the past several months in rodents (Kurle, Koch, Tershy, & Croll, 2014). Abundance of ^15^N increase with transfer between trophic levels relative to ^14^N, and the ratio of these stable isotopes (δ^15^N) can be used to define an organism's trophic position. Due to the difference in assimilation of stable isotopes of carbon by primary producers in ecosystems, ratio of carbon isotopes (δ^13^C) can be used as an indicator of the food source (Ben‐David & Flaherty, 2012). Samples of bank vole fur and putative food items (collected from the vole sample locations) were analyzed for δ^15^N and δ^13^C (Appendix S1 and Table S2). Fur δ^15^N values were correlated with gene expression changes (TMM‐normalized count values of all annotated DEGs in both tissues) using Pearson correlations in r 3.5.0 (function ‘rcorr’; R Core Team, 2018), with *p*‐values corrected by Benjamini‐Hochberg method and considered significant at FDR < 0.05.

## 3 RESULTS

### 3.1 Bank vole body condition

All individuals were adult females with no significant difference in either weight (g, mean ± *SD*: Vesnyane = 22.4 ± 4.8, Gluboke = 21.8 ± 3.0, Kyiv west = 21.0 ± 2.6, Kyiv east = 22.2 ± 3.3; ANOVA, *F* = 0.319, *df* = 3, *p* = .812) or head width, a proxy for age (Kallio et al., 2014) (mm, mean ± *SD*: Vesnyane = 13.0 ± 0.4, Gluboke = 12.8 ± 0.5, Kyiv west = 13.1 ± 0.3, Kyiv east = 12.8 ± 0.2; ANOVA *F* = 1.109, *df* = 3, *p* = .356) among the four sampling locations. Animals from the CEZ and Kyiv area did not differ significantly in body condition index (BCI, mean ± *SD*: Vesnyane = 0.09 ± 1.3, Gluboke = 0.16 ± 0.9, Kyiv west = −0.56 ± 0.5, Kyiv east = 0.31 ± 0.9; ANOVA *F* = 1.565, *df* = 3, *p* = .215).

### 3.2 De novo transcriptome assembly and annotation

De novo assembly resulted in 445,192 transcripts (contigs) that clustered in 273,880 ‘genes’ (clusters of contigs traced from the same De Bruijn graph during Trinity assembly), with a transcript N50 = 2,258 bp and E90N50 = 2,874 bp (Table 1). Mapping read data against this transcriptome yielded an overall alignment rate of 93%, with 85% of reads aligning as proper pairs. BUSCO analysis of the combined liver‐spleen transcriptome identified a comprehensive transcriptome, with 80% complete mammalian BUSCOs. Furthermore, 11% of BUSCOs were fragmented, while only 9% were missing. blastx search against SwissProt found 10,376 uniproteins that are represented by near full‐length transcripts (>80% alignment coverage) in the transcriptome (Figure S3). A total of 128,907 transcripts (31,517 ‘genes’) were annotated with at least one GO term leveraged from the top blastx hit(s) (Dryad Data file 1, file 2, file 3).

**Table 1:** Transcriptome assembly statistics

Number of transcripts	445,192
Number of genes	273,880
GC%	48.10
Median contig length	453
Average contig length	1,050.39
N50	2,258
Number of transcripts in E90 set	51,173
E90N50	2,874

### 3.3 Variation in liver and spleen gene expression associated with inhabiting the CEZ

A total of 444 differentially expressed genes (DEGs; fold change ≥2, FDR <0.001) were identified in the livers of bank voles, with 292 genes being upregulated in the CEZ voles and 152 downregulated (Table S3, Dryad Data file 4a). Of these, 188 genes were successfully annotated (i.e., receiving at least one GO term annotation) to a best matching protein with a known function (Swiss‐Prot 2017_04). In spleens, 97 genes were identified as DE, of which were 61 upregulated and 36 downregulated in the voles from the CEZ (Table S3, Dryad Data file 4b), and with 62 genes obtaining at least one GO term. The gene expression profiles cluster by treatment i.e. the contaminated and control areas; hence, variation in transcriptional activity is associated with variation in the radiation levels (Figure S4). Within the CEZ, liver tissue profiles show higher individual variation (Vesnyane and Gluboke comparison with 40 up‐ and 176 downregulated DE genes, Dryad Data file 5) compared to spleen tissue (two nonannotated DE genes).

**Figure 1:**
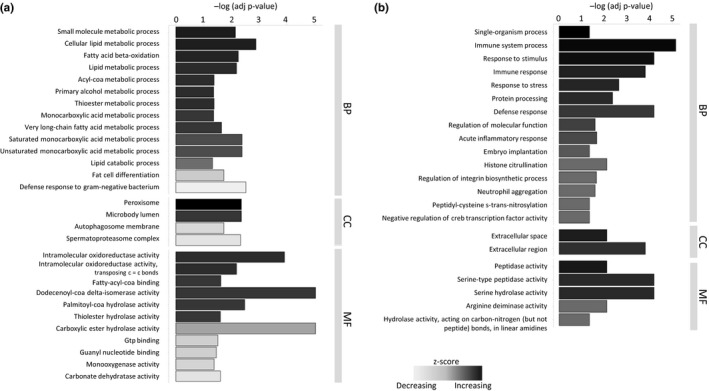
Summarized list of significantly enriched GO terms (FDR < 0.05) among DEGs between uncontaminated Kyiv control and CEZ populations for (a) liver and (b) spleen. GOseq results have been further reduced in REVIGO to remove overlap. GO terms are ordered by category. BP, biological pathway; CC, cell compartment; MF, molecular function and by Z‐score, which indicates the general direction of expression difference of a group of genes containing a given GO term

From the differentially expressed genes, the most significantly enriched GO terms in the biological pathway category in the liver tissue of animals from CEZ were related to lipid metabolic processes, more specifically catabolic processes such as fatty acid beta‐oxidation, metabolic processing of fatty acids and retinols, as well as the acyl‐CoA metabolic processing. Repressed pathways (downregulated DEGs) in the liver include fat cell differentiation and defence responses against bacteria and protozoa. Additionally, immune processes such as cytokine signalling related pathways, antigen processing and virus defence pathways were repressed (Figure 1a, Table S4a,b, Figure S5a,b). The enriched pathways in spleens of animals from CEZ were associated with immune system processes, defence and stress responses and acute inflammatory responses, as well as processing of proteins, e.g., citrullination (Figure 1b, Table S4c). GO term analysis between the two Chernobyl sites showed additional enrichment of peroxisomal activation processes in the genes upregulated in Gluboke liver tissue (See Dryad Data file 5 for GO analysis results).

### 3.4 Metabolic pathways

**Figure 2:**
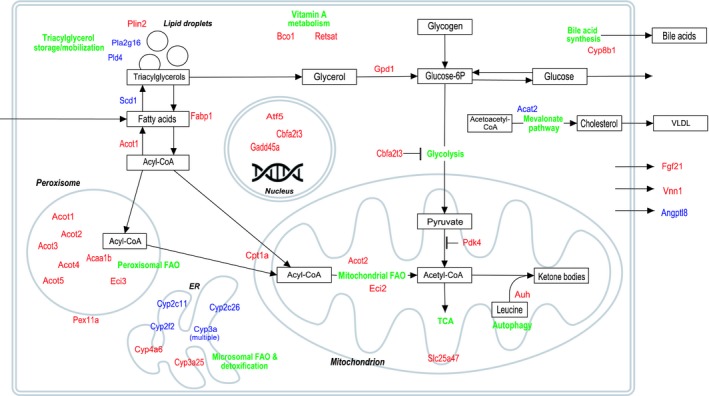
Overview of affected liver metabolic pathways between uncontaminated control and CEZ populations of bank voles. Gene names in red colour indicate upregulation and blue colour downregulation in bank voles of the CEZ compared to control voles. ER, endoplasmic reticulum; FAO, fatty acid oxidation; VLDL, very‐low‐density lipoprotein

Primary energy metabolism pathways were affected in the livers of voles from the CEZ (Table 2). In particular, genes typically associated with mitochondrial fatty acid oxidation (FAO) and accelerated peroxisomal activity were induced (Figure 2), indicating a shift to the use of fatty acids (FA) as a primary energy source. Notable genes that were upregulated include *Cpt1a* (mitochondrial importer of FA; Kersten, 2014), *Pdk4* (a regulator of metabolic flexibility; Zhang, Hulver, McMillan, Cline, & Gilbert, 2014), *Gpd1* (enables gluconeogenesis from glycerol), and *Fgf21* (coordinator of FA utilization, secreted as a hormone by the liver; Fisher & Maratos‐Flier, 2016), which together can promote FAO and gluconeogenesis, while inhibiting oxidation of glucose derived pyruvates in the liver (Fisher & Maratos‐Flier, 2016; Kersten, 2014). Additionally, mitochondrial *Auh*, a central gene of the leucine degradation pathway, where the ketogenic amino acid is converted to ketones, was upregulated. A common theme among these upregulated key metabolic genes is transcriptional control by peroxisomal proliferator activated receptor alpha (PPARα), the master regulator of FAO (Kersten, 2014). While the catabolic processes of FAO were induced in the liver tissue of CEZ voles, a key gene in the FA biosynthetic pathways, *Scd1*, was downregulated. This pattern supports the observed change in fatty acid metabolism, since *Scd1* repression is linked to increased FAO (Paton & Ntambi, 2009). The pattern of elevated FAO was observed in both CEZ sites, with Gluboke voles showing additional upregulation of peroxisomal activity (associated with processing of very long chain fatty acids, see Dryad Data file 5). In the spleen, some genes induced by the PPARγ (peroxisome proliferator‐activated receptors) signalling pathway involved in fatty acid and glucose metabolisms were upregulated in bank voles from Chernobyl (Table 2), but their specific functions in the spleen are unknown.

**Table 2:** Differentially expressed genes in the liver and spleen. Positive fold change values indicate upregulation in bank voles inhabiting the CEZ. PPARa in parenthesis indicate known target of peroxisomal proliferator activated receptor alpha

Gene	Function/description	Fold change (log2)
Liver		
Metabolism and stress		
*Acot1‐5*	Acyl‐CoA thioesterases, peroxisomal FAO, regulation of FA/CoA levels (PPARa)	~1.30
*Fgf21*	Fibroblast growth factor 21, fasting hormone (PPARa)	2.40
*Eci3*	Enoyl‐CoA delta isomerase 3, peroxisomal FAO, very long chain fatty acids	2.24
*Cbfa2t3*	Inhibition of glycolysis. TF	2.05
*Rgn*	Regucalcin, vitamin C synthesis, Ca2+ homeostasis	1.82
*Plin2*	Perilipin‐2, intracellular lipid accumulation marker (PPARa)	1.77
*Eci2*	Enoyl‐CoA delta isomerase 2, mitochondrial FAO (PPARa)	1.67
*Nedd4*	E3 ubiquitin‐protein ligase	1.59
*Cyp4a6*	Cytochrome P450, lipid metabolism	1.58
*G0s2*	G0/G1 switch protein 2, apoptosis, adipogenesis (PPARa)	1.51
*Cyp3a25*	Cytochrome P450, drug & lipid metabolism	1.45
*Gpd1*	Glycerol‐3‐phosphate dehydrogenase, enables gluconeogenesis from glycerol	1.45
*Cyp8b1*	Cytochrome P450, bile acid synthesis (PPARa)	1.43
*Pdk4*	Pyruvate dehydrogenase kinase, regulation of FAO, glycolysis and gluconeogenesis by inhibition of PDC (PPARa)	1.41
*Retsat*	Retinoid (vitamin A derivate) metabolism (PPARa)	1.34
*Cdk3*	Cyclin‐dependent kinase 3, cell cycle regulation	1.31
*Vnn1*	Pantetheinase, vitamin B5 metabolism, AO activity (PPARa)	1.27
*Pex11a*	Peroxisomal membrane protein, peroxisome proliferation	1.23
*Atf5*	Hepatic stress response TF	1.20
*Acaa1b*	Peroxisomal FAO (PPARa)	1.18
*Gadd45a*	Growth arrest and DNA damage‐inducible protein, DNA repair, response to DNA damage	1.16
*Cpt1a*	Carnitine O‐palmitoyltransferase, mitochondrial FAO, rate‐limiting (PPARa)	1.14
*Bco1*	Retinoid (vitamin A derivate) metabolism	1.14
*Pycr1*	Proline synthesis, mitochondrial, OS response	1.14
*Fabp1*	Fatty acid binding protein, binding and transport of intracellular FA, AO activity (PPARa)	1.13
*Slc25a47*	Respiratory uncoupling, mitochondrial	1.10
*Auh*	Methylglutaconyl‐CoA hydratase, mitochondrial, AA degradation (ketogenic)	1.07
*Pla2g16*	Regulation of adipocyte lipolysis (phospholipids), lipid metabolism	–1.00
*Acat2*	Acetyl‐CoA acetyltransferase, mevalonate pathway (cholesterol biosynthesis)	–1.10
*Cyp2c11*	Cytochrome P450	–1.14
*Angptl8*	Angiopoietin‐like protein 8, regulation of serum TAG levels	–1.16
*Pld4*	Phospholipase, phospholipid synthesis	–1.17
*Cyp2c26*	Cytochrome P450	–1.22
*Lpl*	Lipoprotein lipase, cholesterol homeostasis	–1.25
*Cyp2f2*	Cytochrome P450, drug metabolism	–1.27
*Cyp3a family*	Cytochrome P450, various members, drug & lipid metabolism	~ –1.40
*Scd1*	Stearoyl‐CoA desaturase 1, MUFA synthesis, rate‐limiting (PPARa)	–1.85
Immune response		
*Irgm1*	Immunity‐related GTPase, IFNg induced, innate immunity	–1.02
*Tap1*	Antigen peptide transporter 1, antigen processing (MHC‐I), adaptive immunity	–1.07
*Iigp1*	Interferon‐inducible GTPase, resistance to intracellular pathogens, innate immunity	–1.07
*Gstm4*	Glutathione S‐transferase	–1.14
*Psmb8*	Proteasome subunit, antigen processing (MHC‐I), immunity	–1.23
*Batf2*	TF, immune responses	–1.25
*Stat1*	JAK‐STAT cascade, response to IFNs, antiviral	–1.27
*Rnf213*	E3 ubiquitin‐protein ligase	–1.28
*Nlrc5*	Innate immunity, antiviral	–1.32
*Psmb9*	Proteasome subunit, antigen processing (MHC‐I), immunity	–1.40
*Tgtp2*	Innate immunity, resistance to intracellular pathogens, T‐cell‐specific	–1.42
*Ccl19*	Cytokine, chemokine receptor binding, inflammatory response	–1.43
*Socs1*	JAK‐STAT cascade, IFNg signaling	–1.44
*H2 MHC‐I*	Histocompatibility antigens, antigen presenting	–1.46
*Cfhr1*	Regulation of complement cascade, immunity	–1.81
Spleen		
Immune response		
*Mrgprb2*	Mas‐related G‐protein coupled receptor	1.64
*Mcpt8*	Mast cell protease 8	1.48
*Ms4a2*	High affinity immunoglobulin epsilon receptor subunit beta, mast cell activation, IgE binding	1.36
*Cpa3*	Mast cell carboxypeptidase A, angiotensin metabolism	1.35
*Padi4*	Transcription regulation, innate immune responses	1.30
*Cma1*	Mast cell chymase, protease	1.28
*Kng1*	Kininogen, inflammatory response (vasodilation)	1.25
*Prg2*	Proteoglycan, cytotoxin, helminthotoxin, antiparasitic, immunity	1.23
*Mmp9*	Matrix metalloproteinase‐9, inflammatory response, tissue remodeling	1.21
*Spdya*	Speedy protein A, cell cycle regulation, response to DNA damage	1.14
*Hp*	Haptoglobin, antimicrobial, AO, immunity	1.11
*S100A9*	Innate immunity, inflammatory response, antimicrobial, AO, apoptosis	1.11
*Lcn2*	Neutrophil gelatinase‐associated lipocalin, antimicrobial, AO, innate immunity	1.11
*Mcemp1*	Mast cell‐expressed membrane protein 1	1.10
*Alox5*	Arachidonate 5‐lipoxygenase, leukotriene metabolism, inflammatory response	1.08
*Pglyrp1*	Peptidoglycan recognition protein 1, antimicrobial, innate immune response	1.07
*Cebpb*	TF in immune and inflammatory responses	–1.01
*Ube2q2*	Ubiquitin‐conjugating enzyme E2 Q2, protein ubiquitination	–1.01
*Zxdb*	Transcription regulation (MHC class I and MHC class II genes)	–1.03
*Zfp91*	E3 ubiquitin‐protein ligase	–1.69
*Znrf2*	E3 ubiquitin‐protein ligase, antigen processing	–2.04
Metabolism		
*Pdk4*	Regulation of FAO, glycolysis and gluconeogenesis	1.57
*Fabp4*	Fatty acid binding protein 4	1.47
*Plin5*	Lipid droplet protein perilipin	1.27
*Angl4*	Angiopoietin‐related protein 4	1.22

Abbreviations: AA, amino acid; AO, antioxidant; FA, fatty acid; FAO, fatty acid oxidation; MUFA, monounsaturated fatty acid; OS, oxidative stress; PDC, pyruvate dehydrogenase complex; TAG, triacylglycerol; TF, transcription factor.

### 3.5 Response of the innate and active immune system

Genes involved with the innate immune response via granulocytes such as mast cells, neutrophils, eosinophils and basophils, were upregulated in the spleen tissue of voles from the CEZ (Table 2). Genes associated with the release of proinflammatory mediators were upregulated (e.g., *Cma1* chymase, Table 2). For example, upregulation of the lipoxygenase *Lox5* implies increased production of proinflammatory lipid leukotrienes (Caughey, 2007; Metz et al., 2007). Inflammatory reaction was also supported by the upregulation of *Mmp9*, which is involved with mast cell degranulation and inflammation‐associated tissue remodelling processes (Caughey, 2007; Heissig et al., 2005). Activation of innate immune responses was further supported by upregulation of genes involved in pathogen defence responses, such as antimicrobial peptides (*Lcn2, S100A9*) (Levy, 2004), and *Padi4*, which is associated with defence against helminths and other extracellular pathogens (Li et al., 2010). Additionally, genes related to protein ubiquitination, a process involved in regulation of immune responses and degradation of damaged proteins were downregulated in the spleen tissue of voles from the CEZ. For example, the two E3 ubiquitin protein ligases (*Znrf2* and *Zfp91*) are also involved in regulation of adaptive immune responses, such as MHC‐I antigen presenting (Gao & Karin, 2005). Regulation of adaptive immunity responses might be compromised in the spleen tissue by lowered antibody‐producing activity of B‐cells, since expression of some immunoglobulin heavy and light chains was repressed (Table S3).

In contrast to upregulation of some innate immune response pathways in the spleen, key components of the adaptive immune pathway regulating the activity of MHC‐I and antigen processing were downregulated in the livers of bank voles from the CEZ. For example, there was downregulation of immune proteasome genes (*Psmb9* and *Psmb8*), class I histocompatibility antigens and *Tap1,* a transporter protein involved the antigen processing (Kincaid et al., 2012). Additionally, parts of the Janus kinase/signal transducers and activators of transcription, JAK‐STAT signalling pathway (*Stat1* and *Socs1*) (Gao, Wang, Lafdil, & Feng, 2012), were also downregulated in livers of animals from the CEZ.

### 3.6 Cellular response to stress

Bank voles inhabiting the CEZ upregulated transcription of some genes induced by oxidative stress in the liver. For example, mitochondrial oxidative stress response genes were upregulated (e.g., *Atf5*, mitochondrial *Slc25a47, Pycr1* [Fiorese et al., 2016; Kuo et al., 2016; Tan, Ooi, Aw, & Hui, 2004]) and there was an increased activation of genes involved in metabolism of extracellular antioxidants (vitamins B5, A and C) in voles from the CEZ. In the spleen, *Hp* for haptoglobin, an antioxidant that enhances cells' tolerance to oxidative stress (Gutteridge, 1995), was upregulated. In addition, upregulation of two genes generally induced by genotoxic stress or DNA damage was observed (*Gadd45a* in the liver and *Spdya* in the spleen (Liebermann & Hoffman, 2008; McAndrew, Gastwirt, & Donoghue, 2009)), although there were no signs of increased DNA repair efforts, such as increased expression of genes involved in repair of DNA damage, in either tissue. Transcriptional repression of cytochrome P450 genes, such as few members of Cyp2 and several Cyp3a isoforms (families involved in detoxification reactions) was seen in the livers of voles inhabiting Chernobyl.

### 3.7 Gene coexpression networks

**Figure 3:**
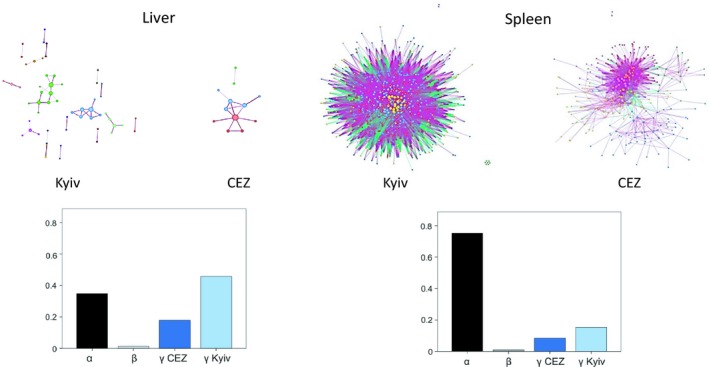
Gene coexpression networks for liver and spleen. The consensus network data is filtered with *p*‐value (.001) and contains only links with high correlation (wTO values for liver: 0.44 for CEZ and 0.47 for Kyiv; wTO for spleen: 0.70 for CEZ and 0.72 for Kyiv). Networks were clustered with the Louvain algorithm, and the clusters are colour‐coded. The colour of the links represents the sign of the interaction, with purple links being positive and green links being negative interactions. The barplots show the proportion of categories of links^†^ when networks from Kyiv and the CEZ were compared. CoDiNA identified 179,133 links and 2,863 nodes in the liver network, whereas 1,841,074 links and 2,960 nodes were detected in the spleen network †α links, in both networks with the same sign (negative or positive correlation), β links, in both networks but with a different sign; γ links, specific to one network

Inhabiting the CEZ affected the interactions among genes in both tissues, with a general pattern of lower amount of coexpressed gene connections in the networks of the CEZ animals. In the spleen, five densely connected clusters of coexpressed genes with high significant correlation (*p < *.001, and see wto correlation values in Figure 3) were identified in the networks from the samples from uncontaminated areas (Kyiv), whereas fewer genes and links (connections between two genes) were identified in the networks from CEZ. In the liver, gene networks in animals from Kyiv contained more links and clustered gene groups than the networks from the CEZ samples (17 and 5 clusters, respectively; Figure 3). When the coexpression networks from CEZ and Kyiv liver tissues were compared, more than half of the connections among the genes were assigned to either Kyiv or CEZ network (γ links), not shared by both (α links), and high frequency of gene correlation patterns specific especially to Kyiv control voles were observed (Figure 3). Genes with strong correlation patterns in the Kyiv network were associated with pathways of energy metabolism, response to stress, antibiotic metabolism, mitochondrion organization, ROS metabolism and antigen presenting (including DEGs: innate immune response genes *Irgm1, Iigp1* & *Tgtp2*, and MHC‐I genes such as *Stat1* and the proteasome genes), whereas the CEZ animals' networks included genes related to energy metabolism processes (including DEGs: vitamin metabolism genes *Rgn, Vnn1, Restat* and FAO genes *Cpt1a* and *Gpd1* [Figure S6a,b]). Comparatively, in the spleen, most gene coexpression patterns were shared between the treatments (α links) (Figure 3). Highly connected genes in both treatments were involved in RNA and cellular metabolism processes, with some additional immune response genes specific to Kyiv network (DEGs such as the antimicrobial *s100a9* and *Prg2*) (Figure S7a,b). The differentially expressed genes were not strongly represented in the networks of either tissue. However, a pattern of lower amount of coexpression patterns among genes (links) in the CEZ voles compared to Kyiv networks was visible in both tissues (Figure 3, Figure S8).

### 3.8 Stable isotopes

**Figure 4:**
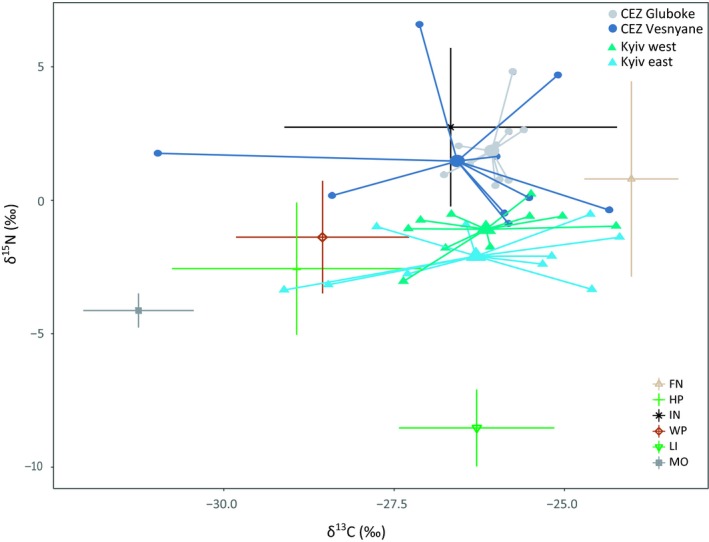
Carbon (δ^13^C) vs. nitrogen (δ^15^N) isotopic values for the fur samples of *Myodes glareolus* inhabiting the CEZ and uncontaminated locations near Kyiv (with mean values of each group in the centre), and their potential dietary sources. Fur isotopic values were corrected downwards (by factors of 2.2‰ for δ^13^C and 2.8‰ for δ^15^N, see Kurle et al., 2014) to account for differences between isotopic values of animal tissue and food sources. Dietary sources^‡^ are presented with means and *SD* ^‡^FN, fungi; HP, herbaceous plants; IN, insects; LI, lichens; MO, mosses; WP, woody plants

Carbon stable isotope values (δ^13^C) had wide interindividual variation at all locations except at Gluboke from CEZ, where marked interindividual homogeneity was observed (Figure 4); hence, there was significant difference in the variance of δ^13^C among groups (Levene's *F*‐statistics, *F* = 4.92, *df* = 3,36, *p* = .006). There was no significant difference in variance in values of δ^15^N among sampling sites (Levene's *F*‐statistics, *F* = 2.76, *df* = 3,36, *p* = .056), albeit with high variation present among individuals from Vesnyane (Figure 4, Table S5). Mean δ^15^N values were significantly higher in the animals from CEZ compared with animals from the uncontaminated sites (Kruskal–Wallis test, *χ* ^2^ = 27.70, *df* = 3, *p* < .001; Table S6 for pairwise tests), with the mean δ^15^N being on average 3.22‰ greater in the samples of fur from animals inhabiting the CEZ. There was no significant difference in the mean δ^13^C values among sampling sites (Kruskal–Wallis test, *χ* ^2^ = 0.035, *df* = 3, *p* = 1.000). In general, bank vole diets from Kyiv animals place in between fungi and woody & herbaceous plants in the dual isotope space, whereas voles from the CEZ were placed closer to insects (Figure 4). In the liver tissue, the expression levels of nearly half of the DEGs (107 out of 188 annotated DEGs) correlate with δ^15^N. The positive correlation with many of metabolically relevant genes related to induction of fatty acid oxidation, such as *Cpt1a*, *Pdk4* and *Fabp1* (Table S7), suggest that the affected metabolic pathways are largely associated with an apparent increase in fur nitrogen isotope levels in the CEZ animals.

## 4 DISCUSSION

Detrimental effects of exposure to radionuclides have been reported at multiple biological scales in wildlife, with elevated levels of DNA damage and oxidative stress as the common characteristic associated biomarkers. Counter to our predictions, the major gene pathways associated with exposure to radionuclides in bank voles inhabiting the CEZ were related to fatty acid metabolism, consistent with an increase in stable isotope values of nitrogen in samples of bank vole fur. We also note that bank voles inhabiting the CEZ show changes in immune response and inflammatory pathways.

### 4.1 Low dose IR is associated with altered metabolic pathways in the liver

Gene expression profiles of bank voles inhabiting the CEZ associate with maintenance of a metabolic state with induced fatty acid oxidation (FAO; Table 2), with the impacted pathways controlled largely by PPARα. PPARα is typically activated by a prolonged negative energy balance (for example starvation or fasting) or ketogenic (low carbohydrate, high fat) diet (Kersten, 2014). Energy stress exhausts liver glycogen, after which fatty acids and glycerol are expected to be mobilized from body fats (triacylglycerols) for energy use. Subsequently, most tissues can switch to FAO as a main energy source via *Cpt1a* upregulation to increase mitochondrial import of Acyl‐CoA (Figure 2), as is observed in the livers of voles inhabiting the CEZ (Table 2).

However, alterations of lipid metabolism can have wider impacts on organismal bioenergetics. When FAO is promoted, the liver typically initiates gluconeogenesis and the production of ketone bodies. However, support for these processes in our data was ambiguous: upregulation of *Gpd1* and *Pdk4* can promote both gluconeogenesis and FAO, and increased leucine degradation can contribute to increased ketone levels (IJlst et al., 2002), though no further support for ketogenesis was seen (Table 2, Figure 2). One consequence of failure to induce ketogenesis or gluconeogenesis in response to an increase in FAO is insufficient resources for the brain, as the brain disfavours long chain fatty acids (Schönfeld & Reiser, 2013) and requires glucose or ketones as a source of energy. With this in mind, there is a negative correlation between brain size and radionuclide levels in birds nesting within the CEZ (Møller et al., 2011). Direct measurements of whether brain size or developmental processes are impacted by metabolic alterations or direct exposure effects in bank voles and other species from the contaminated areas within the CEZ are needed.

An important but unresolved issue is whether the increased FAO in the CEZ bank voles is a direct consequence of exposure to radiation (and possibly adaptive in animals exposed to radionuclides) or represents a more passive indirect response to differences in habitat within and outside the CEZ. In addition, the enrichment (>3‰) of fur nitrogen isotope values (δ^15^N) associated with inhabiting the CEZ is fairly high, i.e., comparable to a shift in trophic level (e.g., Ben‐David & Flaherty, 2012). Elevated radionuclide levels are associated with altered community structure and a reduction in biodiversity (Geras'kin, Fesenko, & Alexakhin, 2008), raising the possibility that altered metabolism reflects some change in habitat or diet associated with bank voles inhabiting the CEZ. Some support for an altered diet might be derived from the different gastrointestinal microbiota communities in bank voles from areas contaminated by radionuclides compared with animals from uncontaminated areas (Lavrinienko et al., 2018). Bank voles have a diverse diet (Butet & Delettre, 2011), and the elevated nitrogen isotope levels in the CEZ animals could arise from an increase in consumption of food with higher δ^15^N, such as invertebrates, fungi or seeds (Calandra et al., 2015). However, a reduction in the abundance of invertebrates in areas with high levels of environmental radioactivity within the CEZ (Møller & Mousseau, 2018) argues against a more invertebrate‐rich diet in animals from the CEZ. In general, the large variation in dietary carbon indicates that bank voles exploit a diversity of plant resources (Calandra et al., 2015), with no apparent dietary difference among our sample areas.

Besides diet, another explanation for the nitrogen enrichment in bank voles inhabiting the CEZ is induction of metabolic pathways associated with catabolic processes, and this is consistent with the transcriptional signature of increased FAO. Generally, nutritional stress (such as starvation or fasting) increases δ^15^N in tissues and fur due to catabolism (Hobson, Alisauskas, & Clark, 1993; Petzke, Fuller, & Metges, 2010). Despite the metabolic signals associated with nutritional stress, neither weight loss nor poor body condition characterize the voles from the CEZ, suggesting that the bank voles inhabiting the CEZ are not starving or deprived of food. Hence, maintaining a metabolic state with increased FAO without obvious loss of body weight can be a part of a strategy to cope with a stressful environment. An increase of FAO as a response to environmental pollution is not commonly reported, although some evidence of altered lipid metabolism can be found in the Atlantic cod (*Gadus morhua*) exposed to methylmercury (Yadetie et al., 2013). Also, exposure to depleted Uranium and gamma rays caused activation of PPARα in the Atlantic salmon (*Salmo salar*) (Song et al., 2016). Altered expression of PPARα‐associated genes is also linked with metabolic diseases (Seo et al., 2008). Nevertheless, a metabolic switch from glycolysis towards FAO might also be beneficial as an antitumoural strategy within an oxidative environment as tumours commonly depend on glycolysis‐related anabolic pathways to support their growth (Vander Heiden, Cantley, & Thompson, 2009).

### 4.2 DNA repair and oxidative stress response

Given that increased DNA damage such as strand breaks is associated with exposure to radionuclides (reviewed by Lourenço et al., 2016), it is interesting that bank voles inhabiting the CEZ exhibited few signs of increased DNA repair activity (see Table 2). However, the altered metabolic pathways may have wider influence here, as both energetic and cellular stress may boost DNA repair pathways, decrease age‐related oxidative stress (Heydari, Unnikrishnan, Lucente, & Richardson, 2007), promote catabolic pathways (such as FAO) and repress growth signalling pathways while promoting cell survival (Yuan, Xiong, & Guan, 2013) and genomic stability. Elevated expression of cell cycle regulators can also increase resistance to oxidative and genotoxic stress as well as to starvation in *Drosophila* (Moskalev et al., 2012). Indeed, fibroblast cells isolated from bank voles from the CEZ show increased resistance to cell death against DNA damaging agents, and can more efficiently recover after irradiation (acute high dose of 10 Gy) (Mustonen et al., 2018), contrary to a pattern of premature senescence and loss of proliferation ability commonly seen in cells sensitive to radiation (Loseva et al., 2014). Within the approximately 50 generations of inhabiting the CEZ (Baker et al., 2017), bank voles may have evolved some resistance to radiation induced DNA damage and oxidative stress (see also Galvan et al., 2014), or are not receiving a sufficiently high dose of radiation to inflict a DNA repair response. Although altered telomere homeostasis is a sign of cellular stress in bank voles inhabiting the CEZ (Kesäniemi, Lavrinienko, et al., 2019), no increases in DNA damage (measured as chromosomal aberrations [Rodgers & Baker, 2000]) or mutation rate, i.e., heteroplasmy (Kesäniemi et al., 2018) in bank voles from contaminated CEZ areas have been reported.

Species affected by the Chernobyl nuclear accident show divergent levels of oxidative damage and antioxidant defences (Einor et al., 2016), such as increased glutathione levels in birds inhabiting the CEZ (reviewed by Lourenço et al., 2016). Counter to our expectations of elevated oxidative stress response, glutathione peroxidase or other antioxidant metabolism genes, such as superoxide dismutases (SOD) and catalase (Kam & Banati, 2013; Limón‐Pacheco & Gonsebatt, 2009) were not differentially expressed in bank vole livers or spleens. However, inhabiting the CEZ may enhance the levels of extracellular antioxidants derived from the diet. Genes involved in vitamin metabolism pathways were induced in bank vole livers (Table 2), such as *Vnn1*, which can enhance production of the radioprotective agent cysteamine (Ferreira, Naquet, & Manautou, 2015), and retinoid metabolism related genes, which also act as antioxidants (Shiota, Tsuchiya, & Hoshikawa, 2006). However, interpreting activity of these vitamin metabolism genes in terms of oxidative stress is difficult as they can have pleiotropic effects on key pathways such as fatty acid metabolism and inflammation (Naquet, Giessner, & Galland, 2016; Shiota et al., 2006). Elevated levels of oxidative stress can damage mitochondria, leading to further increased ROS (reactive oxygen species) production and severe cellular oxidative stress (Kam & Banati, 2013). Therefore, responding to oxidative stress threats and maintaining functional mitochondria is particularly relevant to wildlife within the CEZ.

### 4.3 Tissues differ in their stress response

Based on clinical radiotherapy studies in humans, spleen tissue is considered as more radiosensitive than liver (Rubin & Casarett, 1968). In bank voles, the data suggests that on a transcriptional level, chronic low dose radiation exposure leads to more pronounced stress response in the liver tissue compared to spleen, supported by the higher number of DE genes. Additionally, several processes related to immune responses and lipid metabolism were either repressed or enhanced in the liver. In the spleen, repression of biological processes was limited in voles from the CEZ, and for example, minimal signs of oxidative stress was seen (Table 2). In rodents, radiation exposure induces tissue specific changes also in epigenetic markers (Pogribny, Raiche, Slovack, & Kovalchuk, 2004). Additionally, inhabiting the CEZ changes or disrupts interactions among genes or their regulatory pathways, with treatment specific (CEZ/Kyiv) changes on coregulation more obvious in liver tissue of bank voles, despite its lower suggested radiosensitivity. Previous studies have also shown that coexpressed gene networks are shaped by environmental factors or stressors (Des Marais, Guerrero, Lasky, & Scarpino, 2017; Rose, Seneca, & Palumbi, 2016). As liver and spleen differ in their biological function, differences in their stress responses on a gene expression level is not unexpected. However, immune responses were sensitive to environmental radiation contamination in bank vole liver and spleen tissues, as immune system GO terms were identified in DE genes from both tissues, and the coexpression patterns of genes involved in immune system processes responded to radionuclide contamination. Similarly, pollution induced oxidative stress impacts expression profiles and coregulation of immune response genes in human endothelial cells (Gong et al., 2007).

### 4.4 Immunosuppression in the liver tissue

Exposure to radioactivity affects inflammatory and immune systems in clinical settings (Di Maggio et al., 2015; Frey, Hehlgans, Rödel, & Gaipl, 2015), and our data show comparable processes in wildlife exposed to radionuclides. Immune cells, especially T‐lymphocytes, are sensitive to radiation (Soule et al., 2007) and thus vertebrates exposed to radionuclides typically show immunosuppression and reduced white blood cell (lymphocyte) numbers or a change in the proportion of leucocyte types (Lourenço et al., 2016; Mikryakov, Gudkov, Mikryakov, Pomortseva, & Balabanova, 2013). Immunosuppression (e.g. downregulation of antigen processing pathways) in the livers of bank voles inhabiting the CEZ (Table 2) could imply lowered cellular immunity against intracellular pathogens, such as viruses. For example, MHC‐I and immune proteasomes are vital for lymphocyte mediated antigen presenting and processing (Ferrington & Gregerson, 2012); also, our data are consistent with proteasome function being sensitive to a wide dose range (0.17–20 Gy) of ionizing radiation in mouse and human cell lines (Pajonk & McBride, 2001). The inflammatory response of the liver tissue from bank voles from the CEZ is not consistent with the high dose radiotherapy (single dose of >1 Gy) induced activation of cytokine induced proinflammatory pathways. Bank voles exposed to chronic low dose radiation rather show signs of repression of interferon induced (especially IFNγ) pathways, such as JAK‐STAT signalling, which can lead to lowered antiviral defences (Gao et al., 2012; Rauch, Müller, & Decker, 2013).

### 4.5 Inhabiting the CEZ is associated with activation of granulocytes in spleen tissue

Activation of granulocytes is a common, inflammatory response to radiation in clinical studies (Soule et al., 2007). Accordingly, evidence of inflammation in bank voles inhabiting the CEZ include increased expression of genes involved in activation of mast cells and other granulocytes (Table 2), via the release and synthesis of proinflammatory signalling molecules (such as proteases and leukotrienes, Table 2) (Caughey, 2007; Metz et al., 2007). Mast cells can also release cytokines and be activated by them; however in our data, there was no evidence of differential expression of cytokine genes (Table 2).

Despite the predominantly proinflammatory role of mast cells, favorable anti‐inflammatory functions have also been reported, for example in response to UV‐B irradiation in mouse skin cells (Hart et al., 1998; Metz et al., 2007). Altered expression of mast cells may have fitness consequences as these cells defend against diverse pathogens and parasites (Caughey, 2007) and appear to be more resistant than other immune cells to radiation induced cytotoxicity (Soule et al., 2007). Mast cell activation may be important for wildlife immunity and host‐parasite dynamics. For example, an acute exposure to high radiation (2–5 Gy or above) increases susceptibility to infectious pathogens in birds and mammals (reviewed by Morley, 2012). An increased susceptibility to infection is associated with radiation exposure in humans and fish exposed to radionuclides also in the CEZ (Lourenço et al., 2016; Morley, 2012). Small mammals inhabiting areas of elevated radionuclides had higher ectoparasite burdens and increased prevalence of protozoan parasites (Morley, 2012), and bank voles from contaminated areas within the CEZ had an increased prevalence of helminth infections (Sazykina & Kryshev, 2006). The association of radionuclide exposure and parasites is not well studied, therefore measurements of parasite burdens and blood cell counts are needed to better understand how the suite of impacts associated with suppression and activation of active immunity and mast cells respectively.

To conclude, inhabiting an area with elevated radionuclide contamination affects multiple biological pathways in wild bank voles, highlighting processes associated with lipid metabolism and immune responses in a tissue specific manner. As human actions continue pose new environmental challenges to wildlife, it becomes increasingly important to examine how species react to chronic environmental stressors, such as exposure to radiation or other pollutants.

## AUTHOR CONTRIBUTIONS

J.K., and P.W. designed the experiment, J.K., T.J., A.L., K.K., M.K., T.M., and P.W. performed the research, J.K. and T.J. analyzed the results. J.K., and T.J. wrote the manuscript in collaboration with all authors.

## Data Availability

Additional details on the transcriptome assembly & annotation (files 1–3) and additional results from DESeq and GOseq analyses (files 4–5) are accessible from Dryad Digital Repository (https://doi.org/10.5061/dryad.j3c6r69; Kesäniemi, Jernfors, et al., 2019). Raw transcriptome reads are accessible from Genbank (NCBI SRA SRP15797).
