# Supplementary material for: Exposure to environmental radionuclides is associated with altered metabolic and immunity pathways in a wild rodent
Source: Mol Ecol. 2019 Sep 30;28(20):4620–35. doi: 10.1111/mec.15241 (PMC6900138; doi:10.1111/mec.15241)
Supplement: Supplementary file 1 [file MEC-28-4620-s001.pdf]

MOLECULAR ECOLOGY

Supplemental Information for:

Exposure to environmental radionuclides is associated with altered metabolic and immunity pathways in a wild rodent

Jenni Kesäniemi, Toni Jernfors, Anton Lavrinienko, Kati Kivisaari,
Mikko Kiljunen, Tapio Mappes, Phillip C. Watts

Table of Contents:

Legends for separate SI files	Page 2
Methods	Page 3
Figure S1	Page 5
Figure S2	Page 6
Figure S3	Page 7
Figure S8	Page 7
Table S1	Page 8
Table S2	Page 9
Table S5	Page 9
Table S6	Page 9

Legends for separate Supplemental Information (SI) files

Additional figures as pdf.files:

Figure S4. Tissue specific heatmaps showing the exression patterns of the differentially expressed genes (DEG) when individuals are clustered based on the sample locations, as well as correlation of the gene expression patterns with levels of radiation. Individuals are shown on the y-axis and the DEGs on the x-asis. A) liver, B) spleen (with uniprot gene IDs).

Figure S5. Visualization of GO terms for DEGs A) upregulated and B) downregulated (FDR <0.05) when the CEZ samples and uncontamited Kyiv control samples were compared for the liver tissue. GOseq results have been reduced in REVIGO to remove overlap.

Figure S6. Visualization of GO terms for the genes in the liver tissue gene co-expression network, specifically the CoDiNA comparison between CEZ and Kyiv networks. A) genes specific to CEZ network (γ CEZ nodes), B) genes specific to uncontaminated Kyiv control network (γ Kyiv nodes). Results have been reduced in REVIGO to remove overlap.

Figure S7. Visualization of GO terms for the genes in the spleen tissue gene co-expression network, specifically the CoDiNA comparison between CEZ and Kyiv networks. A) genes specific to CEZ network (γ CEZ nodes) B) genes specific to uncontaminated Kyiv control network (γ Kyiv nodes). Results have been reduced in REVIGO to remove overlap.

Additional tables as excel files:

Table S3. Differentially expressed genes in liver and spleen in CEZ bank voles compared to voles from the Kyiv control areas. RNA sample processing information: RNA extraction dates for each sample.

Table S4. A) GOseq output for Chernobyl-upregulated gene set (compared to Kyiv control) in the liver. B) GOseq output for Chernobyl-downregulated gene set (compared to Kyiv control) in the liver. C) GOseq output for Chernobyl-upregulated gene set (compared to Kyiv control) in the spleen. No significant GO terms were assigned to the Chernobyl-downregulated set of genes for the spleen tissue.

Table S7. Pearson correlations (r.d15N) of differentially expressed genes (DEGs between CEZ and Kyiv, TMM-normalized expression data) and nitrogen stable isotope ($\delta^{15}N$) values (liver and spleen tissues separately).

Methods: additional details on the data analyses

Sequencing, de novo transcriptome assembly, annotation and data analysis

The libraries were sequenced on an Illumina HiSeq4000 (Beijing Institute of Genomics, Hong Kong). Read data were cleaned (*i.e*. removing adapters and low quality reads with QC cutoff=20, and demultiplexing) using SOAPnuke v.1.5.5 (Chen et al., 2018). The transcriptome was assembled using Trinity v.2.4.0 (Grabherr et al., 2011). Following the transcriptome assembly, the assembly read representation was assessed using Bowtie 2 v.2.2.9 (Langmead & Salzberg, 2012). N50 was calculated for the E90 set of transcripts that represent 90% of the total expression data for the assembled samples. Transcript length coverage statistics and the amount of near full-length transcripts were determined by BLAST+ v.2.6.0 (Altschul, Gish, Miller, Myers, & Lipman, 1990) search against Swiss-Prot (release 2017_04). The transcriptome completeness was determined from number of single copy orthologs assessed using BUSCO v.2.0 (Benchmarking Universal Single Copy Ortholog) (Simão, Waterhouse, Ioannidis, Kriventseva, & Zdobnov, 2015). We used a BLASTx search against all known proteins in Swiss-Prot database (release 2017_04) to determine protein length coverage statistics. The transcriptome was annotated using Trinotate v.3.0.1 annotation pipeline (https://trinotate.github.io/).

RNA was extracted from the samples during 4 consequtive days (mixing tissues and samples from different locations). As an example, a PCA plot with sample processing information (8 batches of samples) was done to visualize the effect of sample RNA extraction batch on the clustering of the individual expression profiles (no clustering seen, see figures below). Sequencing was performed using 4 lanes, with 2 lanes for mixed samples of liver and 2 for the spleens.

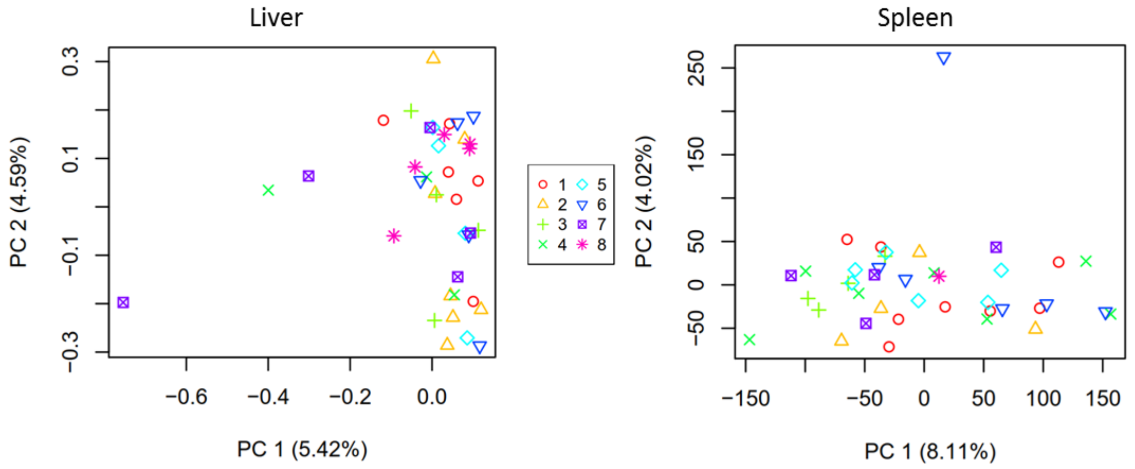

Gene co-expression networks

When co-expression networks from the CEZ and Kyiv were compared using CoDiNA, it also classifies each node (=gene) into a category based on whether the links of the node are distributed equally among the categories (α, present in both networks with the same sign) or whether the links are distributed unequally among the categories (β, present in both networks but with a different sign and γ, specific to one of the networks) (Gysi, de Miranda Fragoso, Buskamp, Almaas, & Nowick, 2018).

In the liver tissue comparison of networks from CEZ and Kyiv, 735 γ CEZ specific nodes, 1468 γ Kyiv specific nodes, 455 α and 1 β node were identified (see Figure S8). In the spleen comparison, most nodes were shared by treatments (2221 α nodes), and 394 γ CEZ nodes, 324 γ Kyiv nodes & 18 β

nodes were identified. GO terms were assigned to the gene lists of the treament specific categories (γ CEZ, γ Kyiv) in both tissues using GOTermFinder (P-value cutoff 0.01) (Boyle et al., 2004) and the results were visualized using REViGO (Supek, Bošnjak, Škunca, & Šmuc, 2011) (see Figures S6 and S7).

Stable isotope analysis

Bank vole fur was clipped from the thoracic area and prepared according to the protocol of Bligh & Dyer (1959) to remove lipids and then dried over night. The dried fur samples (0.5-1.0 mg, packed into tin capsules) were analysed for stable isotopes of carbon and nitrogen using a Thermo Finnigan DELTA^{plus} Advantage CF-IRMS connected to a Carlo Erba Flash EA1112 elemental analyser at the University of Jyväskylä, Finland. Results are expressed using the standard δ notation as parts per thousand (‰) difference from the international standards. The reference materials used were internal standards of known relation to the International Atomic Energy Agency (IAEA) standards of Vienna Pee Dee belemnite (for carbon) and atmospheric N2 (for nitrogen). Dried pike (*Esox lucius* L.) white muscle was used as an internal working standard for the fur samples.

Samples of putative bank vole food sources were sampled opportunistically from the same locations as the bank voles, during 29 May – 02 June, 2018. The samples were stored in paper envelopes at room temperature. All samples were oven dried (at 60°C for 72 h), and homogenised into a powder using steel beads and TissueLyser II (Qiagen) system. Powdered samples were then weighed (0.5-1.2 mg), packed into tin capsules and analysed for stable isotopes of carbon and nitrogen similarly as the fur samples. Dried pike (*Esox lucius* L.) white muscle was used as an internal working standard for insects, lichens, mosses and fungi, whereas birch (*Betula pubescens*) leave sample was used as the internal working standard for the herbaceous and woody plants. In all runs, analytical precision was always better than 0.2‰ for both $\delta^{15}N$ and $\delta^{13}C$, and was determined by repeated analysis of internal working standards [dried pike (*Esox lucius* L.) white muscle tissue and/or birch (*Betula pubescens*) leaves] inserted in to each run after every five samples.

References

Altschul, S. F., Gish, W., Miller, W., Myers, E. W., & Lipman, D. J. (1990). Basic local alignment search tool. *Journal of Molecular Biology*, *215*(3), 403–410. doi:10.1016/S0022-2836(05)80360-2

Blight, E., & Dyer, W. (1959). A rapid method of total lipid extraction and purification. *Canadian Journal of Biochemistry and Physiology*, *37*(8), 911–917. doi:10.1139/o59-099

Boyle, E. I., Weng, S., Gollub, J., Jin, H., Botstein, D., Cherry, J. M., & Sherlock, G. (2004). GO::TermFinder - Open source software for accessing Gene Ontology information and finding significantly enriched Gene Ontology terms associated with a list of genes. *Bioinformatics*, *20*(18), 3710–3715. doi:10.1093/bioinformatics/bth456

Chen, Y., Chen, Y., Shi, C., Huang, Z., Zhang, Y., Li, S., … Chen, Q. (2018). SOAPnuke: A MapReduce Acceleration supported Software for integrated Quality Control and Preprocessing of High-Throughput Sequencing Data. *GigaScience*, *7*, 1–6. doi:10.1093/gigascience/gix120

Grabherr, M. G., Haas, B. J., Yassour, M., Levin, J. Z., Thompson, D. A., Amit, I., … Regev, A. (2011). Full-length transcriptome assembly from RNA-Seq data without a reference genome. *Nature Biotechnology*, *29*(7), 644–52. doi:10.1038/nbt.1883

Gysi, D. M., de Miranda Fragoso, T., Buskamp, V., Almaas, E., & Nowick, K. (2018). Comparing multiple networks using the Co-expression Differential Network Analysis (CoDiNA). *arXiv Preprint arXiv:1802.00828*.

Langmead, B., & Salzberg, S. L. (2012). Fast gapped-read alignment with Bowtie 2. *Nature Methods*, *9*(4), 357–359. doi:10.1038/nmeth.1923

Simão, F. A., Waterhouse, R. M., Ioannidis, P., Kriventseva, E. V., & Zdobnov, E. M. (2015). BUSCO: assessing genome assembly and annotation completeness with single-copy orthologs. *Bioinformatics*, *31*(19), 3210–3212. doi:10.1093/bioinformatics/btv351

Supek, F., Bošnjak, M., Škunca, N., & Šmuc, T. (2011). Revigo summarizes and visualizes long lists of gene ontology terms. *PLoS ONE*, *6*(7). doi:10.1371/journal.pone.0021800

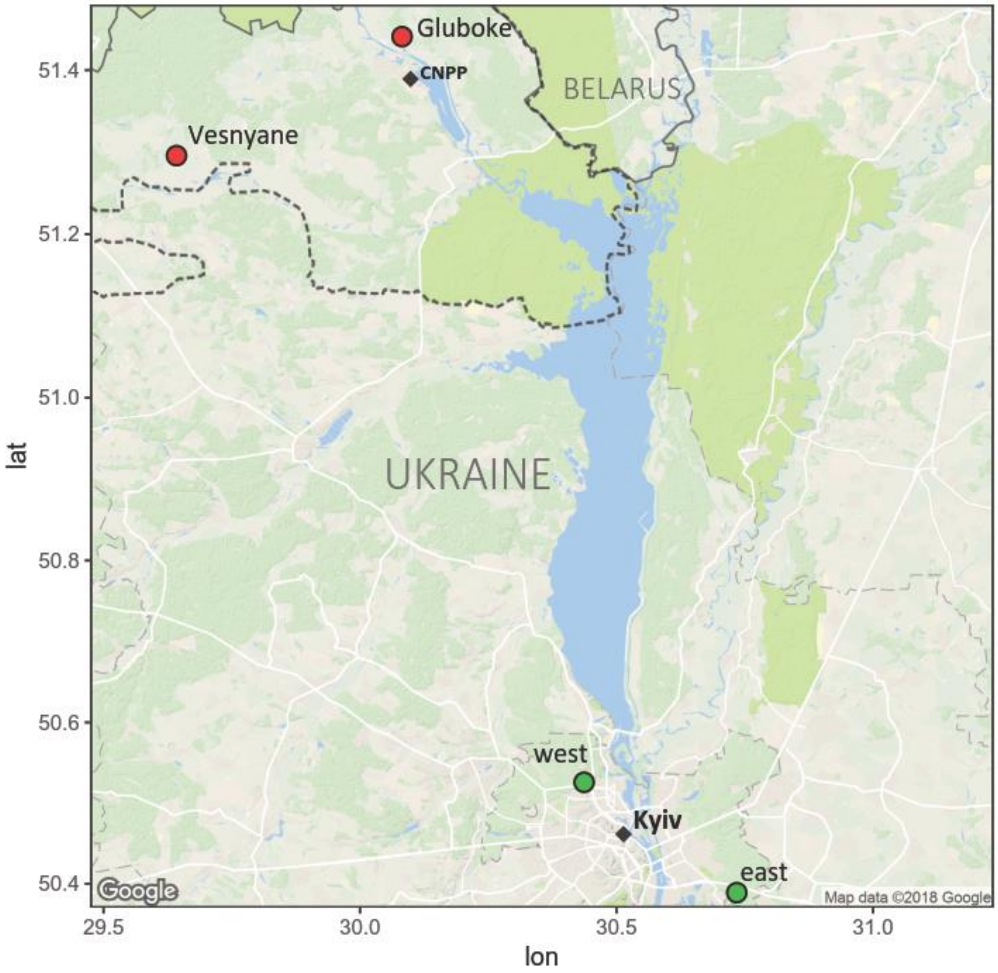

Figure S1. Map of the bank vole sampling locations in Ukraine. The CEZ is marked with a dotted line, and CNPP shows the location of the Chernobyl nuclear power plant (CNPP).

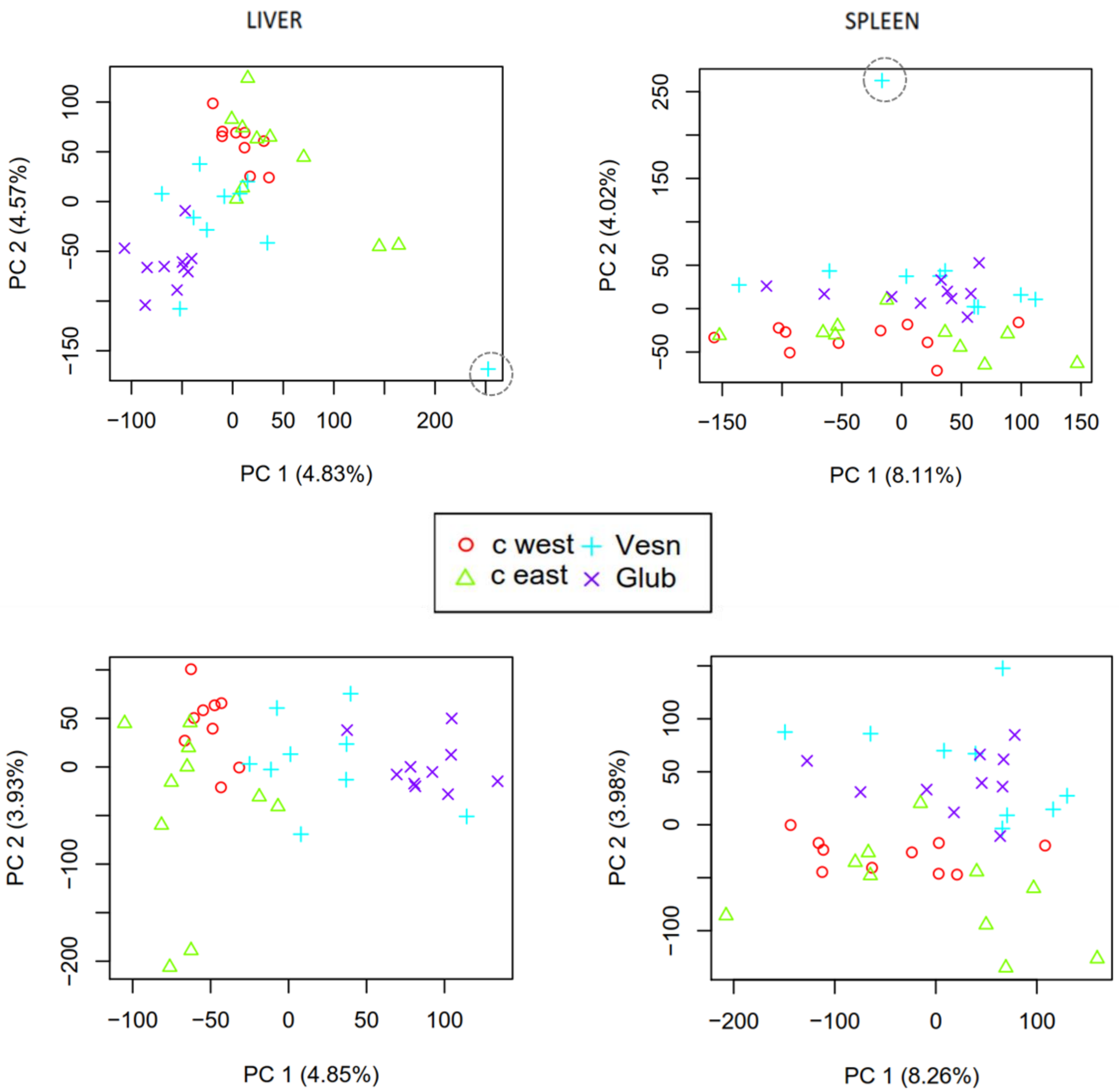

Figure S2. Upper panel: PCA plots of the transcriptome data showing the outlier individual from Vesnyane (circled with a grey dotted line) (c=control site from the Kyiv area). Lower panel: PCA plots of the transcriptome data with the outlier individual removed (this set of samples was used in the downstream analyses).

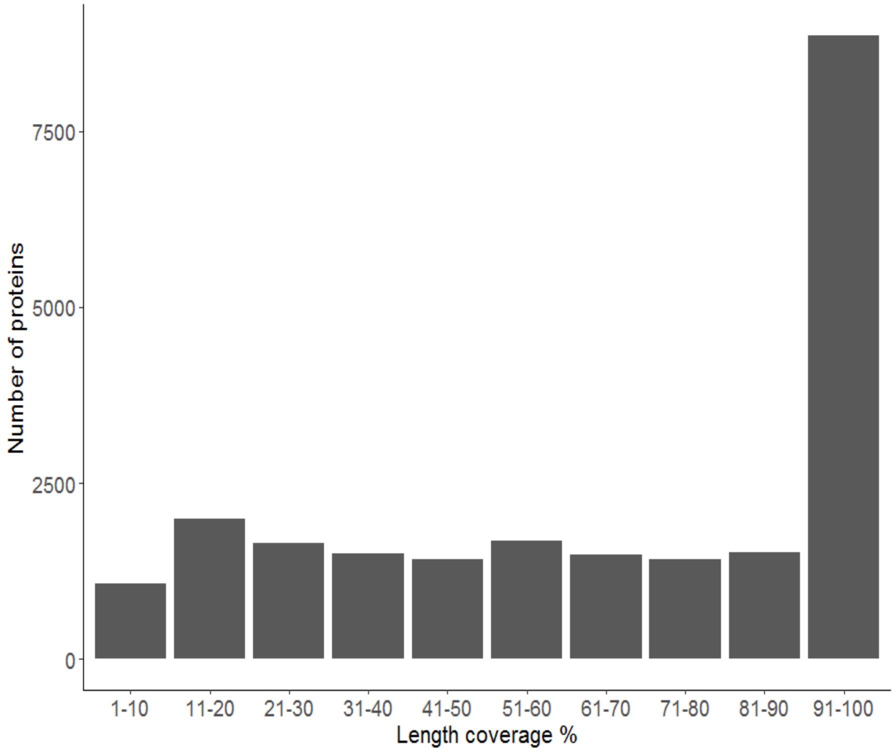

Figure S3. Histogram for the number of uniproteins covered by transcripts in the assembled transcriptome (BLASTx–Swiss-Prot).

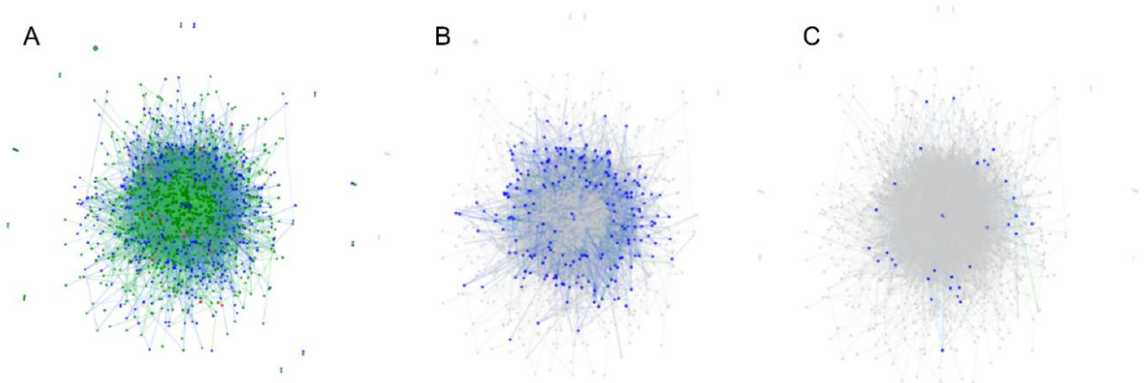

Figure S8. Visualization example of the comparison of the liver gene co-expression networks from Kyiv and CEZ animals. A) Liver CoDiNA network with links and nodes (genes connected by links) shared between Kyiv and CEZ shown in green. B) Kyiv specific links and genes (blue), C) CEZ specific links and genes (blue). Note that not all links appear in the filtered networks (1,303 nodes and 17,678 links illustrated here).

Table S1: Sample library sizes in number of read pairs (after QC). Samples in bold were used in assembly.

Sample	Site	Liver lib.size (PE reads)	Spleen lib.size (PE reads)
74	Kyiv-west	19330715	19610033
75	Kyiv-west	19146243	17676664
78	Kyiv-west	16407516	16562306
81	Kyiv-west	16444631	14885663
85	Kyiv-west	16005203	15629949
90	Kyiv-west	20875075	17238390
93	Kyiv-west	n/a	15052790
**101**	**Kyiv-west**	**19555660**	**17077645**
104	Kyiv-west	18785129	17195085
107	Kyiv-west	15491470	19700710
721	Kyiv-east	17532620	19437074
**722**	**Kyiv-east**	**20457676**	**19946837**
723	Kyiv-east	18308686	17156398
724	Kyiv-east	19643939	17730116
726	Kyiv-east	22026486	17026708
727	Kyiv-east	19564678	16503293
728	Kyiv-east	22408916	15790288
731	Kyiv-east	16856367	17717332
732	Kyiv-east	20099782	14849515
734	Kyiv-east	24730482	19484979
772	Vesnyane	15709085	19804162
839	Vesnyane	17644228	20264258
840	Vesnyane	15592950	20210606
841	Vesnyane	17187379	17006620
842	Vesnyane	15148999	21138827
**844**	**Vesnyane**	**18097063**	**20504126**
845	Vesnyane	15903655	18651333
866	Vesnyane	18242122	20364772
873	Vesnyane	20119142	19102148
897	Vesnyane	21363279	14379694
960	Gluboke	21571434	22428016
961	Gluboke	15983832	23173594
962	Gluboke	17769129	18378636
963	Gluboke	16112018	15584026
964	Gluboke	19134003	20229262
965	Gluboke	18420541	19392343
966	Gluboke	17089402	19282473
967	Gluboke	16541078	18922902
**968**	**Gluboke**	**21389528**	**19449917**
969	Gluboke	20709677	16695279

Table S2. Stable isotope analysis for putative bank vole food sources, with sample sizes (N) and mean values with standard deviations (SD) for carbon ($\delta^{13}C$) and nitrogen isotope values ($\delta^{15}N$) for the dietary source groups.

Source	N	Mean $\delta^{13}C$ (SD)	Mean $\delta^{15}N$ (SD)
herbaceous plants	52	-28.93 (1.83)	-2.51 (2.47)
woody plants	29	-28.55 (1.27)	-1.38 (2.11)
lichens	4	-26.21 (1.30)	-8.85 (1.43)
mosses	3	-31.50 (0.77)	-4.22 (0.76)
fungi	5	-23.95 (0.49)	1.49 (4.63)
insects	26	-26.67 (2.44)	2.74 (2.97)

Table S5: Within group mean values (SD, standard deviation) and variances for the fur carbon ($\delta^{13}C$) and nitrogen isotope values ($\delta^{15}N$).

	Mean $\delta^{13}C$ (SD)	Variance $\delta^{13}C$	Mean $\delta^{15}N$ (SD)	Variance $\delta^{15}N$
**Kyiv east**	-24.00 (1.77)	3.13	0.93 (1.07)	1.15
**Kyiv west**	-23.85 (1.06)	1.12	1.94 (0.90)	0.87
**CEZ Vesnyane**	-24.27 (1.90)	3.63	4.43 (2.42)	5.86
**CEZ Gluboke**	-23.76 (0.38)	0.14	4.86 (1.29)	1.68

Table S6: Pairwise analysis for the fur nitrogen isotope values ($\delta^{15}N$) among the sampling locations (Kruskall-Wallis test with Dunn's post hoc test, with by a Benjamini-Hochberg FDR correction for multiple comparisons). Pairwise chi-square values are shown below diagonal, and P-values (with italics) above diagonal.

	Kyiv east	Kyiv west	CEZ Vesnyane	CEZ Gluboke
**Kyiv east**		*0.143*	*<0.001*	*<0.001*
**Kyiv west**	-1.18		*0.009*	*<0.001*
**CEZ Vesnyane**	-3.65	-2.50		*0.1958*
**CEZ Gluboke**	4.63	3.45	0.86	
