# Supplementary figures and images for: Exposure to environmental radionuclides is associated with altered metabolic and immunity pathways in a wild rodent
Source: Mol Ecol. 2019 Sep 30;28(20):4620–35. doi: 10.1111/mec.15241 (PMC6900138; doi:10.1111/mec.15241)

A

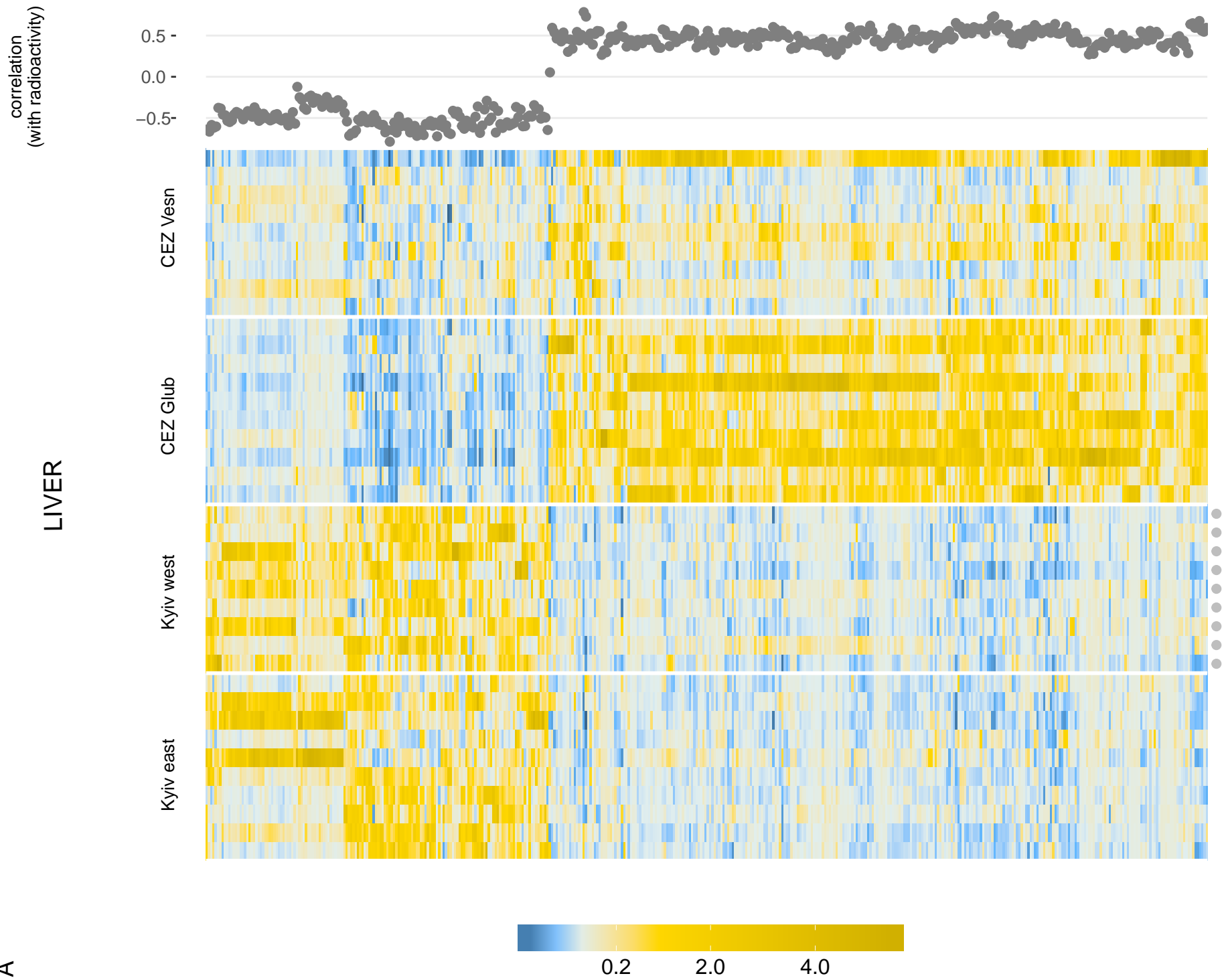

radioactivity  
(log uGy/hr)

0

1

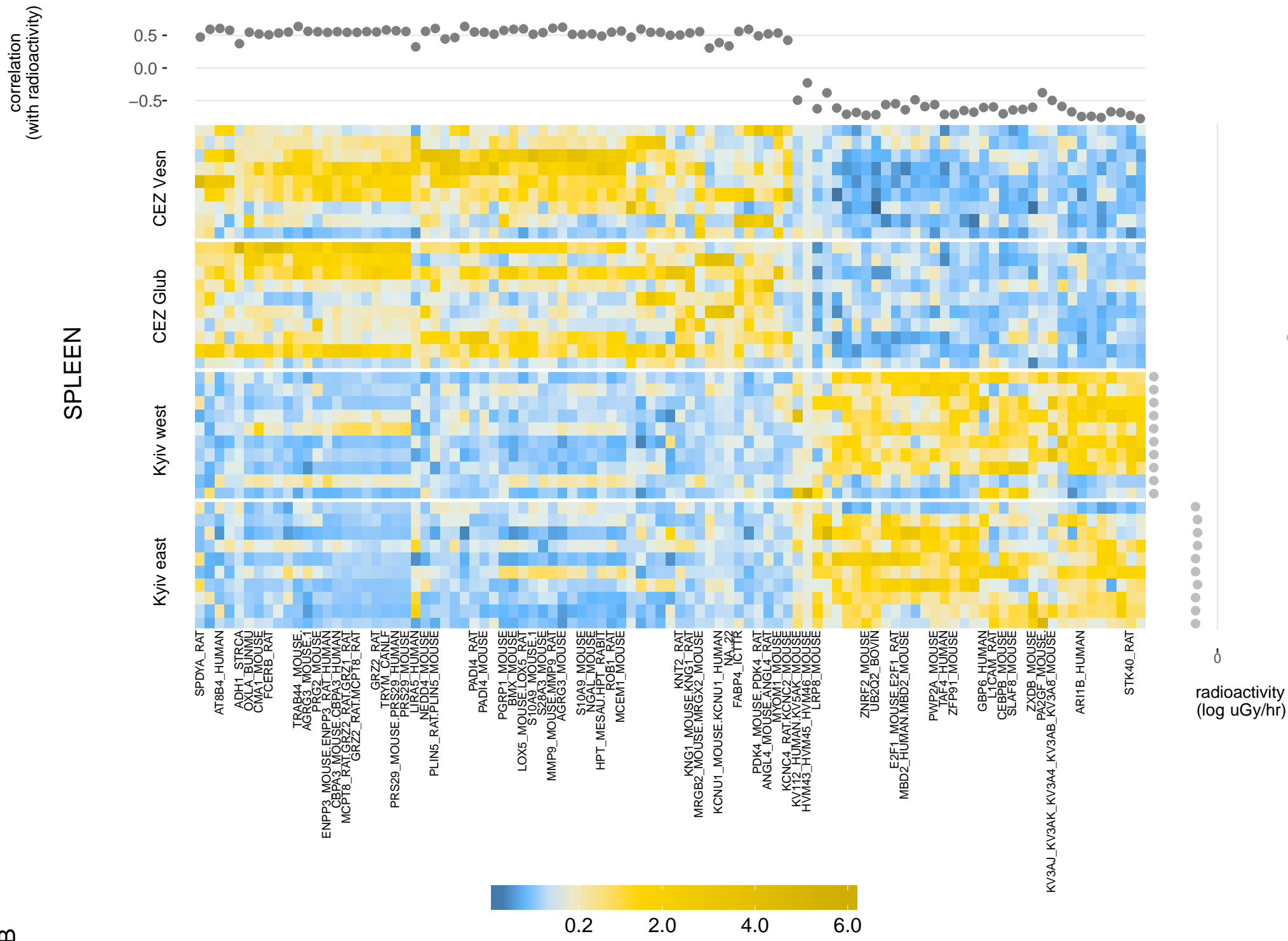

Supplement: Supplementary file 2 [file MEC-28-4620-s002.pdf]
