# Supplementary material for: Exposure to environmental radionuclides is associated with altered metabolic and immunity pathways in a wild rodent
Source: Mol Ecol. 2019 Sep 30;28(20):4620–35. doi: 10.1111/mec.15241 (PMC6900138; doi:10.1111/mec.15241)

A.

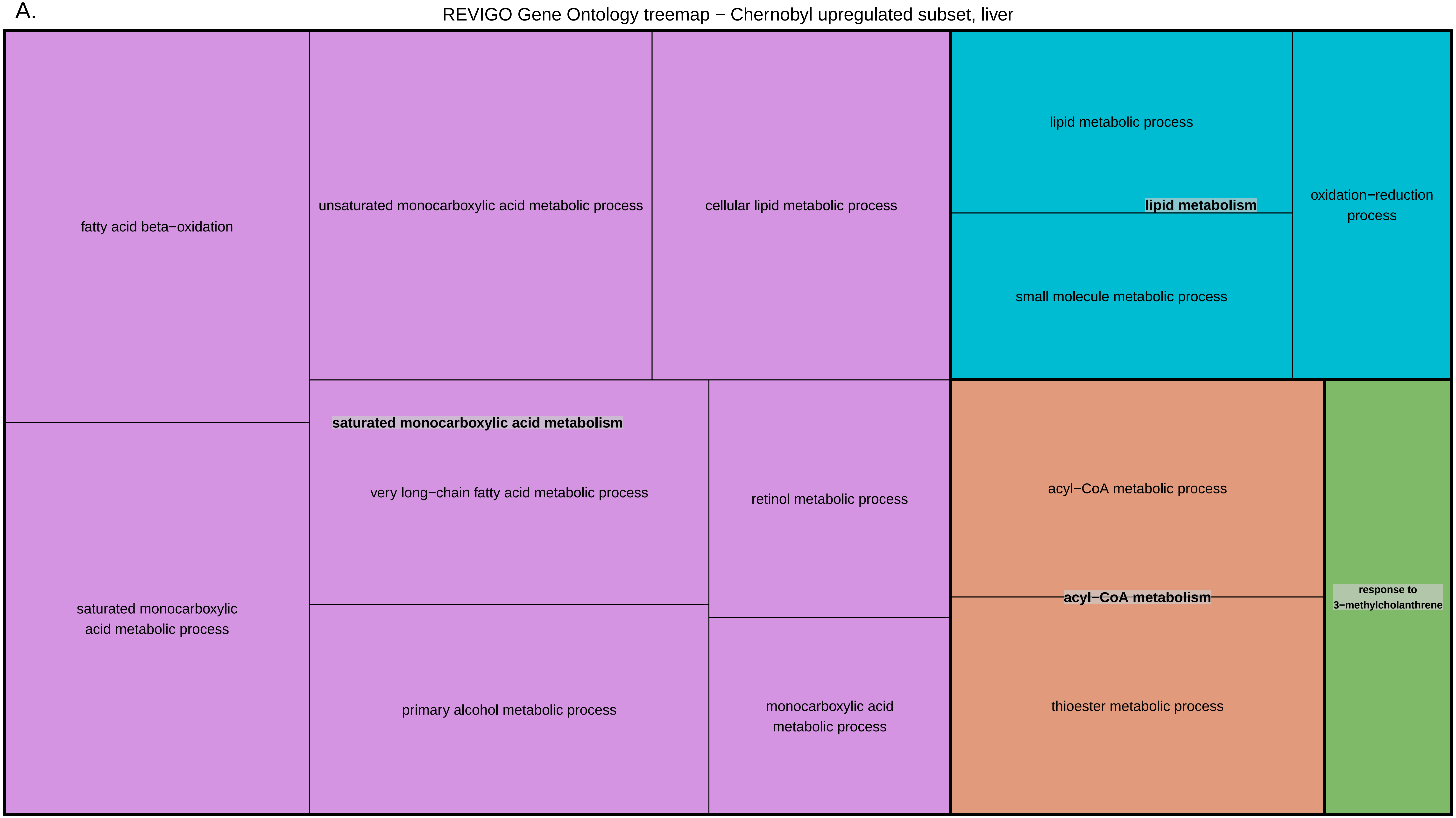
REVIGO Gene Ontology treemap − Chernobyl upregulated subset, liver
fatty acid beta−oxidation
unsaturated monocarboxylic acid metabolic process
cellular lipid metabolic process
lipid metabolic process
lipid metabolism
oxidation−reduction process
small molecule metabolic process
saturated monocarboxylic acid metabolism
very long−chain fatty acid metabolic process
retinol metabolic process
acyl−CoA metabolic process
acyl−CoA metabolism
response to 3−methylcholanthrene
saturated monocarboxylic acid metabolic process
primary alcohol metabolic process
monocarboxylic acid metabolic process
thioester metabolic process

B.

REVIGO Gene Ontology treemap − Chernobyl downregulated subset, liver

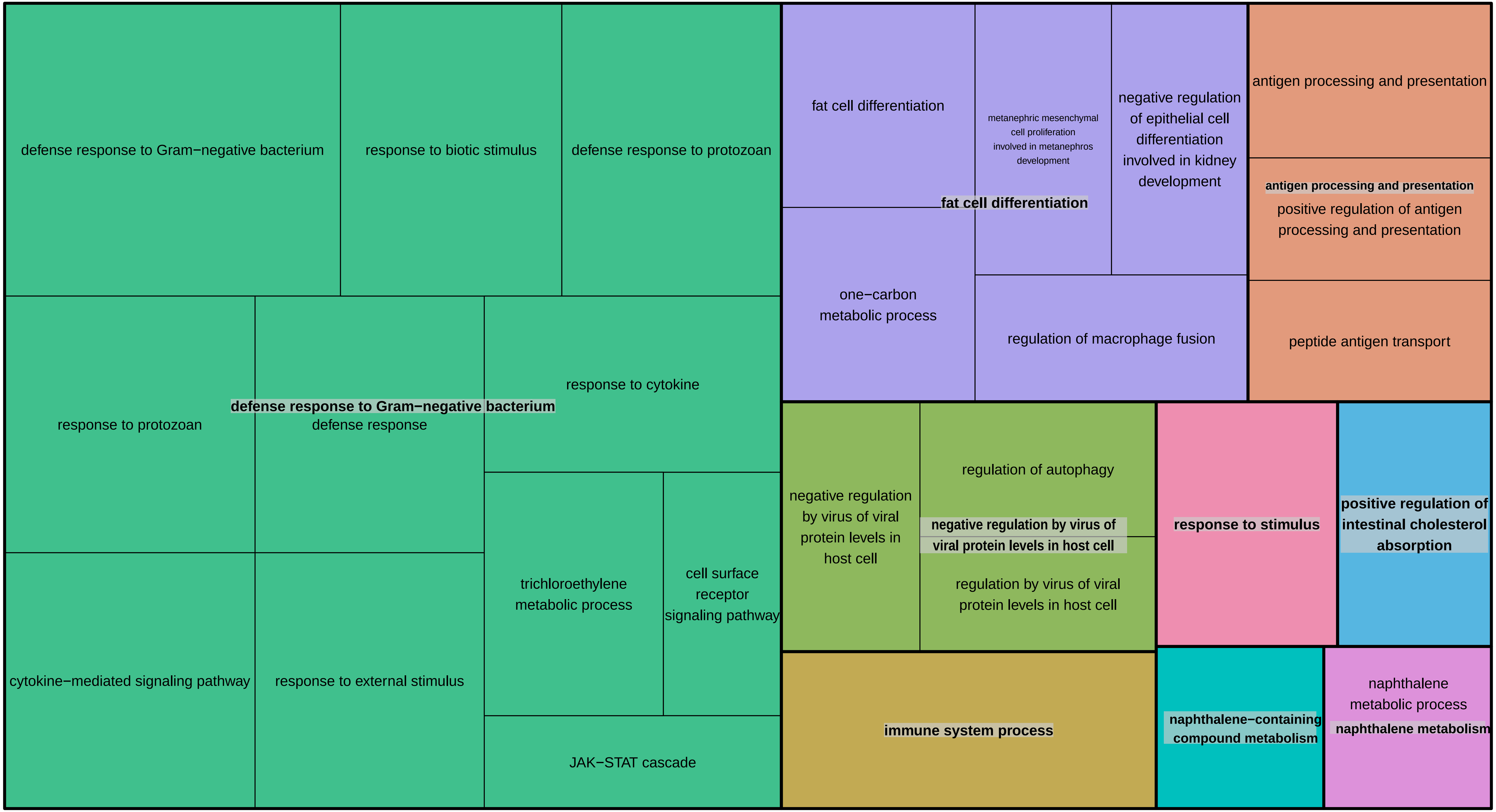

Supplement: Supplementary file 3 [file MEC-28-4620-s003.pdf]
