# Supplementary material for: Exposure to environmental radionuclides is associated with altered metabolic and immunity pathways in a wild rodent
Source: Mol Ecol. 2019 Sep 30;28(20):4620–35. doi: 10.1111/mec.15241 (PMC6900138; doi:10.1111/mec.15241)

A.

REVIGO Gene Ontology treemap - CEZ specific genes, liver (CoDiNA)

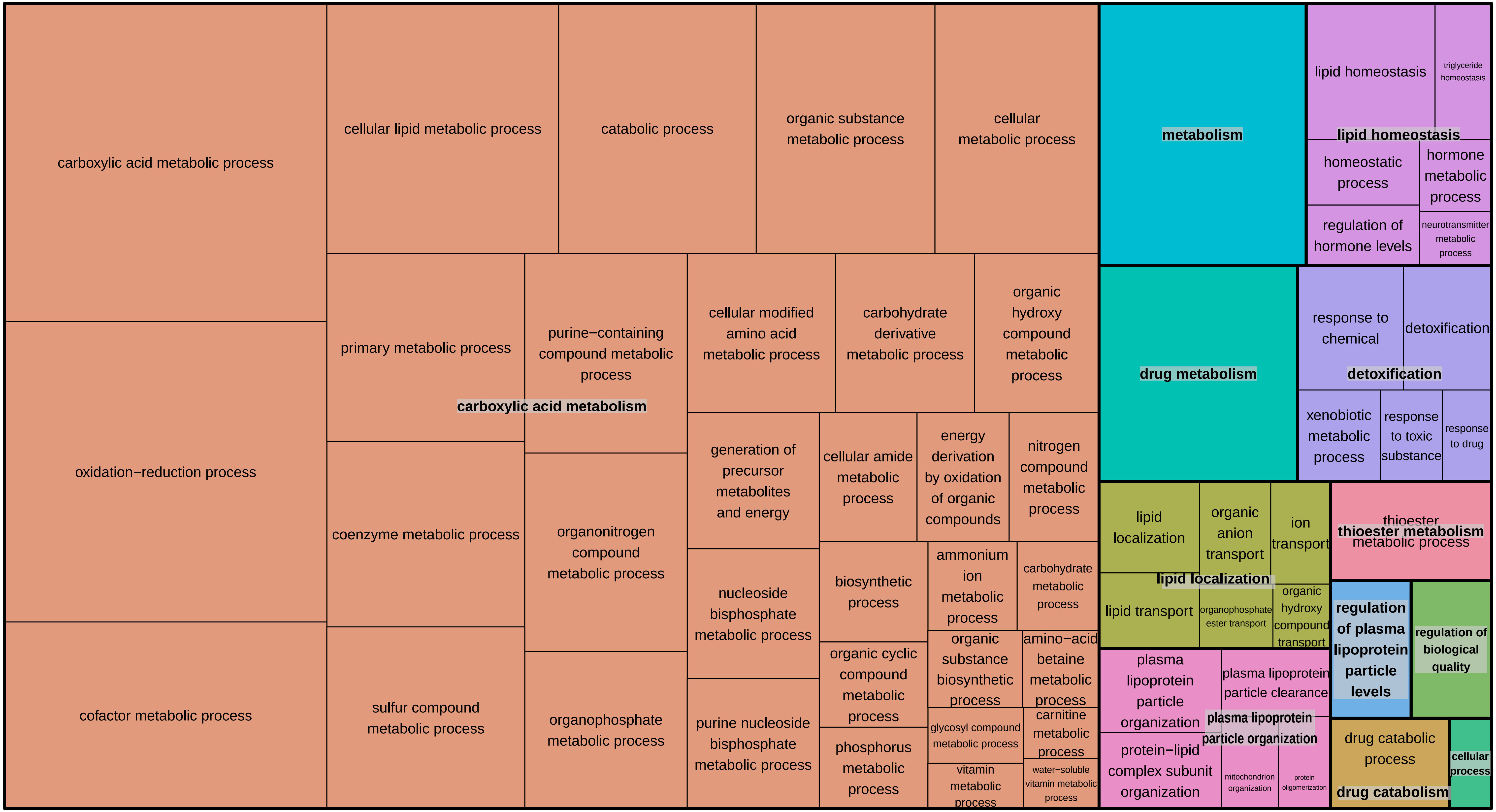

B.

REVIGO Gene Ontology treemap - Kyiv specific genes, liver (CoDiNA)

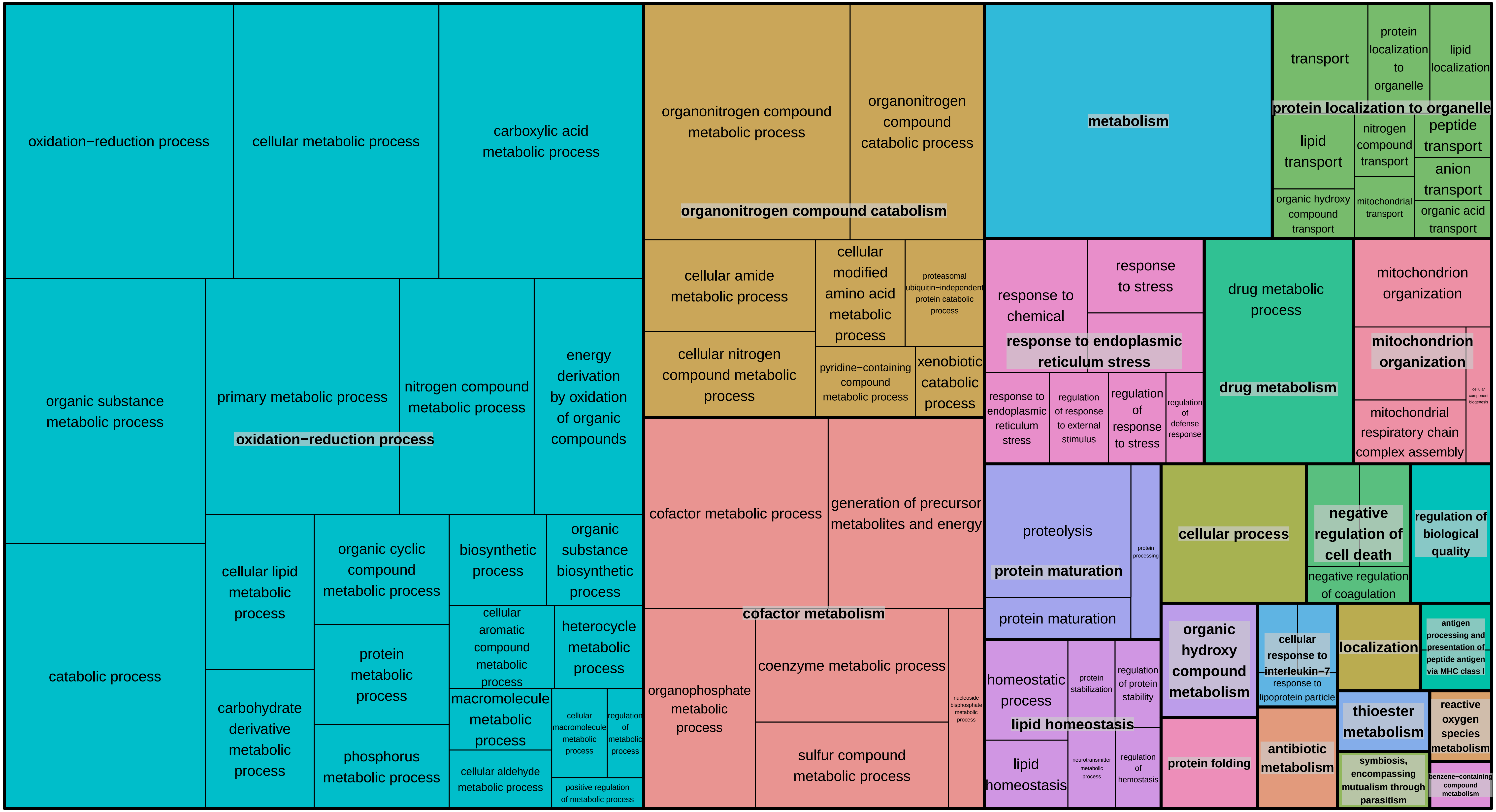

Supplement: Supplementary file 4 [file MEC-28-4620-s004.pdf]
