# Supplementary material for: Exposure to environmental radionuclides is associated with altered metabolic and immunity pathways in a wild rodent
Source: Mol Ecol. 2019 Sep 30;28(20):4620–35. doi: 10.1111/mec.15241 (PMC6900138; doi:10.1111/mec.15241)

A.

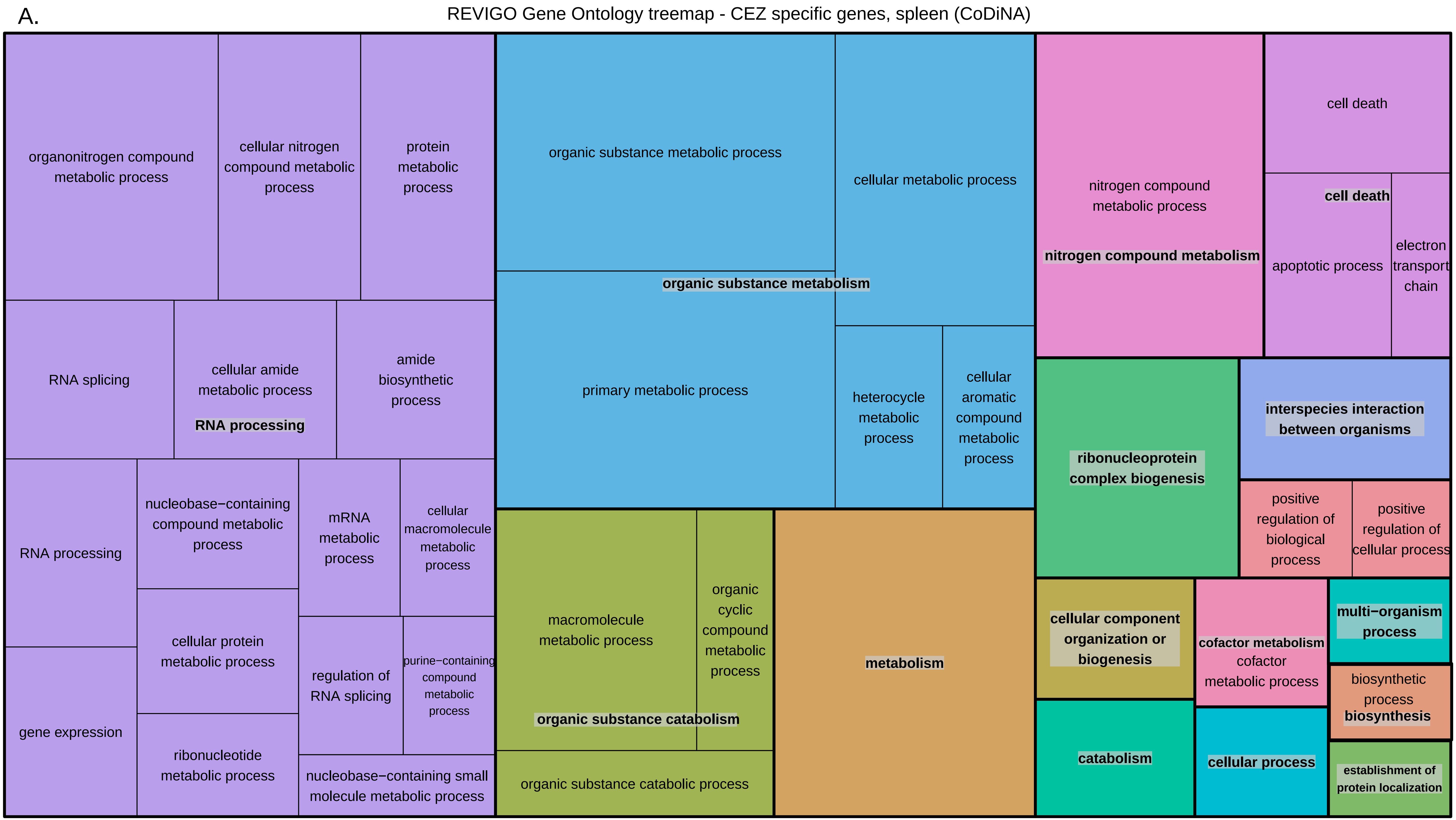
REVIGO Gene Ontology treemap - CEZ specific genes, spleen (CoDiNA)
organonitrogen compound metabolic process
cellular nitrogen compound metabolic process
protein metabolic process
RNA splicing
cellular amide metabolic process
RNA processing
amide biosynthetic process
RNA processing
nucleobase−containing compound metabolic process
mRNA metabolic process
cellular macromolecule metabolic process
cellular protein metabolic process
regulation of RNA splicing
purine−containing compound metabolic process
gene expression
ribonucleotide metabolic process
nucleobase−containing small molecule metabolic process
organic substance metabolic process
cellular metabolic process
organic substance metabolism
primary metabolic process
heterocycle metabolic process
cellular aromatic compound metabolic process
macromolecule metabolic process
organic cyclic compound metabolic process
organic substance catabolism
organic substance catabolic process
metabolism
nitrogen compound metabolic process
nitrogen compound metabolism
cell death
cell death
apoptotic process
electron transport chain
ribonucleoprotein complex biogenesis
interspecies interaction between organisms
positive regulation of biological process
positive regulation of cellular process
cellular component organization or biogenesis
cofactor metabolism
cofactor metabolic process
multi−organism process
biosynthetic process
biosynthesis
catabolism
cellular process
establishment of protein localization

B.

REVIGO Gene Ontology treemap - Kyiv specific genes, spleen (CoDiNA)

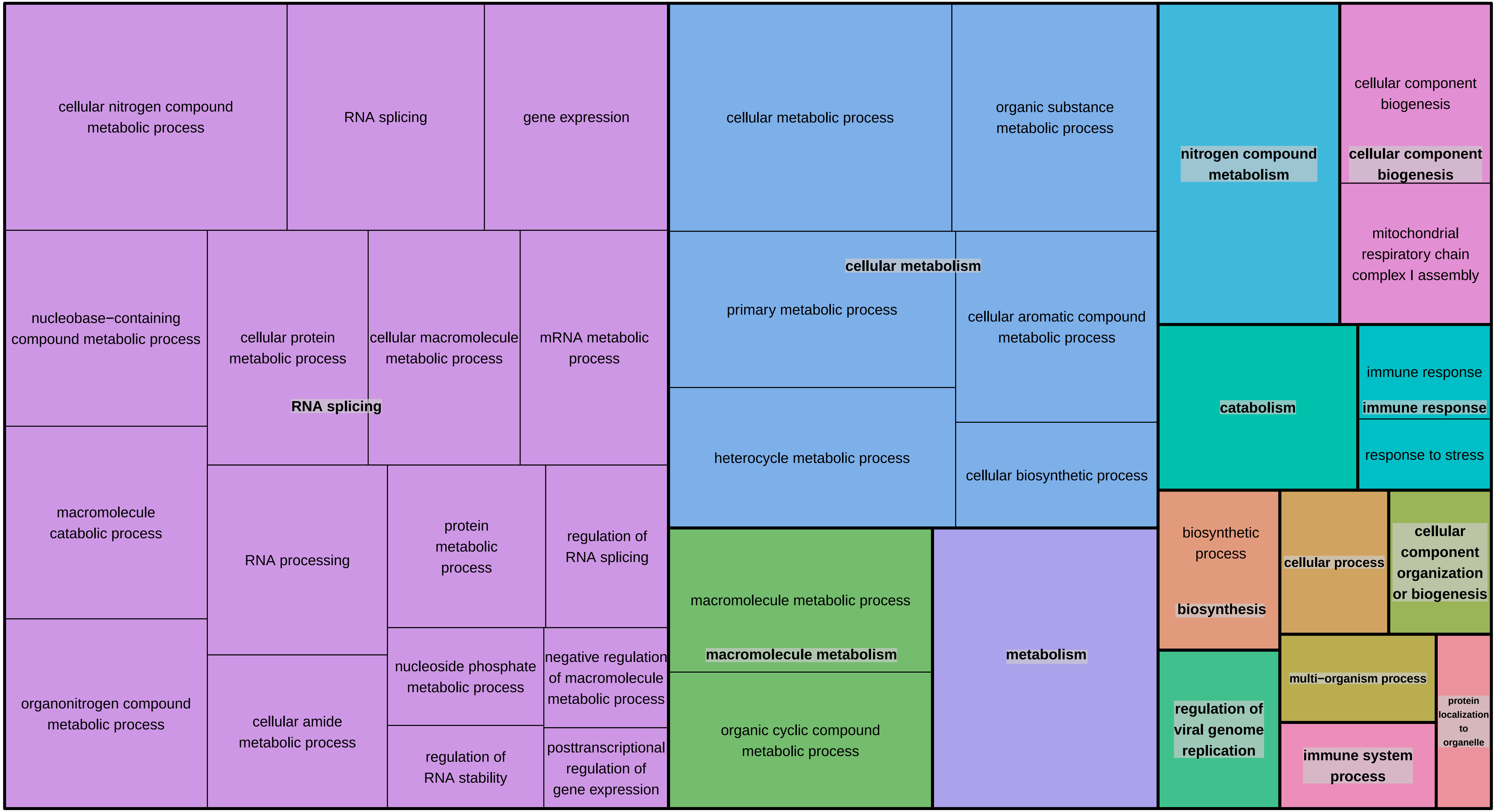

Supplement: Supplementary file 5 [file MEC-28-4620-s005.pdf]
